# Effects of exercise therapy on quality of life in patients with inflammatory bowel disease: a network meta-analysis

**DOI:** 10.3389/fphys.2026.1894474

**Published:** 2026-07-22

**Authors:** Zhiyi Yan, Jing Zhi, Lu Zhao, Shuqing Wang, Keqin Ye, Meilin Yin, Shengsheng Zhang

**Affiliations:** 1 Digestive Disease Center, Beijing Hospital of Traditional ChineseMedicine, Capital Medical University, Beijing, China; 2 Beijing University of Chinese Medicine, Beijing, China

**Keywords:** continuous aerobic exercise, Crohn’s disease, high-intensity interval training, inflammatory bowel disease, network meta-analysis, resistance exercise, Tai Chi, ulcerative colitis

## Abstract

**Objective:**

To systematically evaluate and compare the efficacy of exercise interventions represented in eligible randomized controlled trials in patients with inflammatory bowel disease (IBD), rank the evaluated modalities, and identify factors that may influence treatment outcomes.

**Methods:**

We systematically searched PubMed, Web of Science, Sinomed, Cochrane Library, Embase, CNKI, and Wanfang for randomized controlled trials (RCTs) investigating exercise interventions in patients with IBD. Outcomes included health-related quality of life, disease activity, fatigue, and inflammatory biomarkers. Network meta-analysis (NMA) was conducted using Stata 16.0. For continuous outcomes, pooled effects were expressed as mean differences (MDs) or standardized mean differences (SMDs) with 95% confidence intervals (CIs), as appropriate. Study quality and risk of bias were assessed using RevMan 5.4. Confidence in network estimates was assessed using CINeMA, while certainty for outcomes synthesized by conventional pairwise meta-analysis was assessed using GRADE.

**Results:**

A total of 24 RCTs involving 1,557 patients and six exercise modalities were included. Compared with conventional therapy, continuous aerobic exercise (CAE) significantly improved quality of life (SMD = 1.15, 95% CI [0.29, 2.01]), while high-intensity interval training showed a favorable but imprecise effect. Yoga and high-intensity interval training significantly reduced disease activity, and Yoga also improved fatigue. High-intensity interval training reduced IL-6, whereas CAE and high-intensity interval training were associated with lower TNF-α. Yoga significantly reduced CRP and ESR. Subgroup analyses suggested that continuous aerobic exercise performed four times weekly for three months exhibited a relatively favorable ranking probability within the evidence network for quality-of-life improvement, though this relative position does not imply absolute clinical superiority. The fatigue, IL-6, and TNF-αnetworks contained only subsets of the six exercise modalities, and confidence in many comparisons was low or very low; these estimates and rankings should therefore be interpreted cautiously.

**Conclusion:**

Exercise therapy may improve quality of life and related outcomes in patients with IBD. However, comparative rankings are limited to the modalities represented within each outcome network and are supported predominantly by low- or very-low-confidence evidence; clinical recommendations should therefore remain cautious. Larger, well-designed RCTs are needed to optimize exercise prescriptions.

## 1 Introduction

Inflammatory bowel disease (IBD) is a chronic and recurrent inflammatory illness of the gastrointestinal tract that comprises Crohn’s disease (CD) and ulcerative colitis (UC). It is frequently linked to exhaustion, weight loss, diarrhea, and stomach discomfort (Rubin et al., 2012; Bruner et al., 2023). The global burden of IBD has increased substantially since 2017 and continues to rise, underscoring its growing importance as a global public health challenge (GBD 2017 Inflammatory Bowel Disease Collaborators, 2020; Kaplan and Windsor, 2021). Newly industrialized regions have experienced a rapid rise in incidence. In contrast, high-income regions have entered a phase of compounding prevalence, where relatively stable incidence and low mortality have resulted in a growing number of people living with chronic disease (Kaplan and Windsor, 2021). Recent population-based surveillance further supports this trend, with prevalence reaching as high as 671 per 100,000 population in some heavily affected populations in 2024 (Forbes et al., 2025). Taken together, these findings indicate that IBD is imposing an increasingly substantial and sustained burden on healthcare systems worldwide (GBD 2017 Inflammatory Bowel Disease Collaborators, 2020; Kaplan and Windsor, 2021).

Exercise therapy is a low-risk, modifiable lifestyle intervention that has received increasing attention in the management of chronic diseases and related comorbidities, including chronic kidney disease, depression, breast cancer, coronary artery disease, and functional gastrointestinal disorders (Saud et al., 2021). One of the primary pathogenic mechanisms in IBD is inflammation, and immunological responses are significantly influenced by metabolic control. It is believed that exercise reduces inflammation by causing immune cells to generate anti-inflammatory cytokines and mediators (Mathur and Pedersen, 2008). Consequently, a growing number of randomized controlled trials have examined the effects of structured exercise interventions on various outcomes in patients with IBD, including symptom control, inflammatory markers, and health-related quality of life.

Network meta-analysis (NMA) provides an advantage over conventional pairwise meta-analysis because it allows simultaneous comparisons among multiple interventions, even when direct head-to-head trials are limited. Therefore, this study aimed to synthesize the available evidence on different exercise modalities in patients with IBD. It also compared their associations with quality of life, disease activity, fatigue, and inflammatory biomarkers, and evaluated their relative rankings within the current evidence network.

## 2 Materials and methods

### 2.1 Search strategy

All literature searches were independently conducted and cross-verified by two reviewers (Yan Z.Y. and Zhi J.). Any discrepancies were resolved through discussion with a third reviewer (Zhao L.). A comprehensive search was performed in PubMed, the Cochrane Library, Embase, Web of Science, China National Knowledge Infrastructure (CNKI), Wanfang Academic Journals Full-text Database, and China Biomedical Literature Database (Sinomed), from inception to December 30, 2025. The search terms included “inflammatory bowel disease,” “ulcerative colitis,” “Crohn’s disease,” “exercise therapy,” “yoga,” “continuous aerobic exercise,” “high-intensity interval training,” and “resistance training.” The detailed search strategy is provided in the Electronic **Supplementary Material**. In order to find additional research that satisfied the inclusion criteria, the reference lists of the retrieved papers were also manually examined. The search was intentionally broad and was not restricted to the six exercise categories ultimately represented in the included trials.

Initially, titles and abstracts of all identified studies were screened to exclude irrelevant publications. Subsequently, full texts were assessed for their relevance to outcomes including quality of life, fatigue, disease activity, and inflammatory biomarkers to determine eligibility for inclusion. Study selection was conducted in accordance with the Preferred Reporting Items for Systematic Reviews and Meta-Analyses (PRISMA) statement (Page et al., 2021), and the study protocol was registered in PROSPERO (CRD420261331212).

### 2.2 Inclusion and exclusion criteria

The PICOS framework—which includes Participants, Intervention, Comparison, Outcomes, and Study design—was used to determine the inclusion and exclusion criteria (**Table 1**).

**Table 1:** Inclusion and exclusion criteria based on the PICOS framework.

PICOS element	Inclusion criteria	Exclusion criteria
P (Participants)	Patients aged > 16 years with a clinical diagnosis of Inflammatory Bowel Disease (IBD)	**-**
I (Intervention)	Planned exercise training of any form, intensity, and frequency (see specific classifications in **Table 2**), with an intervention duration of ≥ 4 weeks.	1. Acute studies with an exercise intervention duration < 4 weeks. 2. Studies with a crossover design.
C (Comparison)	Usual care, other non-exercise interventions, or comparisons between different exercise regimens.	**-**
O (Outcomes)	Studies reporting complete data for at least one of the following outcomes: disease activity, fatigue, quality of life, C-reactive protein (CRP), erythrocyte sedimentation rate (ESR), IL-6, TNF-α	Studies from which suitable data for the relevant outcomes cannot be obtained.
S (Study design)	Randomized Controlled Trials (RCTs).	Non-original RCTs such as conference abstracts, study protocols, and systematic reviews.

Exercise is defined as planned, purposeful, and repetitive physical activity. Studies with a crossover design are excluded due to potential carryover effects.

Inclusion criteria: (1) Study design: Randomized controlled trials (RCTs). (2) Participants: Individuals aged over 16 years with a confirmed diagnosis of inflammatory bowel disease (IBD). (3) Interventions: Exercise interventions of any form, intensity, or frequency, with a minimum duration of four weeks. Exercise includes exercise treatments compared to control groups or various forms or intensities of exercise in comparative studies. Six exercise modalities were categorized and defined in **Table 2**. (4) Outcomes: Research that provides comprehensive data for at least one of the following results: disease activity, fatigue, health-related quality of life, C-reactive protein (CRP), erythrocyte sedimentation rate (ESR), interleukin-6 (IL-6), or tumor necrosis factor-α (TNF-α). The six modalities were not prespecified as the only eligible forms of exercise. Rather, they were the modalities represented by the eligible RCTs identified through study selection and were categorized after inclusion according to their principal exercise components. Other approaches, including balance training and progressive muscle relaxation, were not represented among the eligible RCTs included in the quantitative synthesis; their absence should not be interpreted as evidence that they are unsuitable or ineffective for IBD.
Exclusion criteria: (1) Acute studies with intervention duration less than four weeks. (2) Research using a crossover design. (3) Systematic reviews, study protocols, or conference abstracts. (4) Studies for which suitable data were unavailable.

**Table 2:** Definitions of exercise modalities for patients with IBD.

Modality	Definitions
Continuous aerobic exercise	Walking, cycling, and running are examples of continuous exercise that aims to increase the cardiorespiratory system’s capacity and efficiency.
High-intensity interval training	Repetitive relatively short-interval exercise, performed with “all-out effort” or intensity ≥ 90% peak oxygen uptake or heart rate > 90% peak heart rate
Resistance exercise	Exercise designed to increase muscular growth, strength, and endurance
Combined aerobic and resistance exercise	Integrated modality of exercise that combines aerobic exercise and resistance training
Yoga	Ancient Indian systematic fitness routines that enhance respiratory control and focus through breathing exercises and strength, flexibility, coordination, and endurance through postures (asanas)
Tai Chi	An ancient Chinese mind-body practice that enhances balance, coordination, and general well-being via slow, rhythmic motions and deliberate breathing.

CE, Combined Aerobic and Resistance Exercise; CAE, Continuous Aerobic Exercise; TC, Tai Chi; RE: Resistance Exercise; HIT, High-intensity Interval Training.

### 2.3 Study selection process

**Figure 1:**
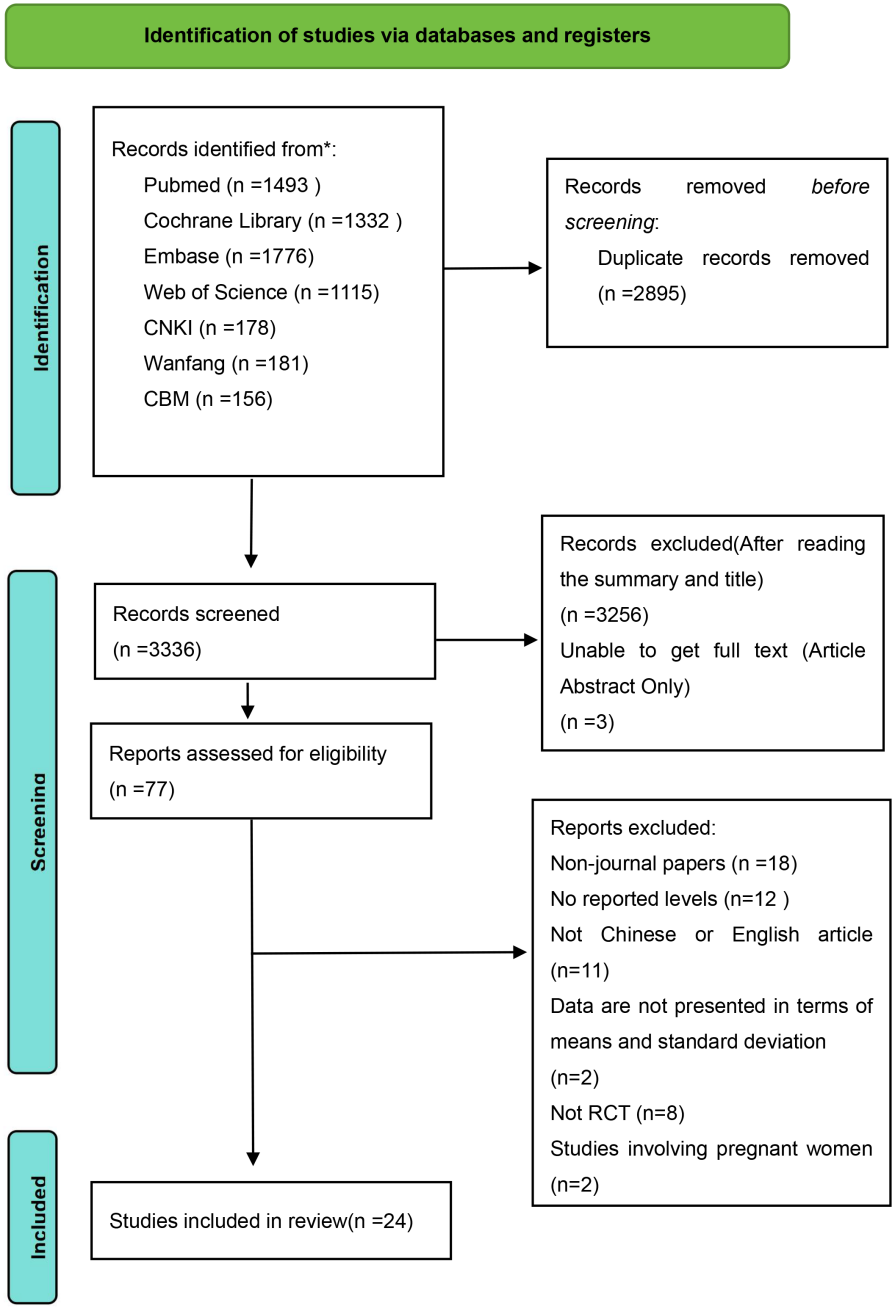
Document screening process and results.

Literature management was performed using EndNote X9. Duplicate studies were eliminated once all collected records were loaded into the program. Titles and abstracts were then independently screened by two reviewers to exclude irrelevant records. In order to verify inclusion, complete texts of possibly eligible papers were acquired and separately evaluated by the same two reviewers. Any disputes were settled by dialogue or, if required, by consulting a third reviewer. A flow diagram (**Figure 1**) of the study selection process shows the grounds for exclusion at each step.

### 2.4 Data extraction

The following information was extracted from each study: study characteristics, including first author, journal, year of publication, randomization method, and blinding; participant characteristics, including sample size, age, sex, and diagnostic criteria for IBD; intervention characteristics; and outcome measures, including disease activity, fatigue, health-related quality of life, CRP, ESR, IL-6, and TNF-α.

### 2.5 Risk of bias assessment

Two reviewers (Yan Z.Y. and Zhi J.) independently assessed the methodological quality of the included randomized controlled trials using the Cochrane Risk of Bias tool in RevMan 5.4 (Higgins and Green, 2011). Seven areas were used to assess bias: (1) random sequence generation; (2) allocation concealment; (3) participant and staff blinding; (4) outcome assessment blinding; (5) incomplete outcome data; (6) selective reporting; and (7) additional causes of bias. Each domain was assigned a bias risk rating of low, uncertain, or high. Any disputes between the two reviewers were settled by discussion; if they were unable to come to an agreement, the third reviewer (Zhao L.) made the decision.

### 2.6 Statistical analysis

#### 2.6.1 Descriptive statistics

Descriptive tables were generated to summarize the basic characteristics of the included studies, including first author, year of publication, diagnostic criteria, intervention duration, exercise type, sample size, age, and sex of participants.

#### 2.6.2 Network meta-analysis and conventional meta-analysis

Network meta-analysis (NMA) and conventional pairwise meta-analyses were performed using Stata 16.0 and Stata 12.0 (network package). Depending on whether the same measurement scale was applied across trials, pooled effects for continuous outcomes were described as mean differences (MDs) or standardized mean differences (SMDs), each with 95% confidence intervals (CIs). Network evidence plots were generated for each outcome using Stata 16.0. To display the outcomes of direct and indirect comparisons between treatments, league tables were created.

The relative efficacy of each exercise modality for each outcome was ranked using the surface under the cumulative ranking curve (SUCRA), with higher SUCRA values suggesting a higher likelihood of being among the best therapies. Because the included outcomes represented different clinical domains, no overall pooled ranking across outcomes was interpreted as definitive.

For outcomes involving few exercise interventions, conventional pairwise meta-analyses were conducted using Stata 12.0. Statistical heterogeneity was assessed with the I²statistic and the corresponding P value. A fixed-effects model was used when I²≤ 50% and *P* ≥ 0.05; otherwise, a random-effects model was applied. Where substantial heterogeneity was detected, pooled estimates were generated using a random-effects model, and subgroup analyses were performed to examine potential sources of heterogeneity. Robustness was assessed through leave-one-out sensitivity analyses, sequentially excluding studies at high risk of bias or predefined subgroups.

#### 2.6.3 Inconsistency assessment

Disagreement between direct and indirect evidence was described as inconsistency. The differences between direct and indirect comparisons inside closed loops were assessed using a loop-specific methodology. Inconsistency factors (IF) were calculated using Stata; an IF value closer to zero indicates better consistency.

#### 2.6.4 Publication bias and sensitivity analysis

Publication bias was examined using comparison-adjusted funnel plots. The horizontal axis represents the difference between the study-specific effect estimate for a given comparison and the pooled estimate from comparable comparisons, while the vertical axis represents the standard error (SE). In the absence of small-study effects, the plot is expected to be symmetrically distributed around the zero line. When more than ten studies were available, Egger’s test was further used to assess potential publication bias. Sensitivity analyses were performed by sequentially excluding one study at a time to examine the robustness of the findings.

#### 2.6.5 Certainty of evidence

Confidence in the network meta-analysis estimates for health-related quality of life, disease activity, fatigue severity, IL-6, and TNF-α was assessed using the Confidence in Network Meta-Analysis (CINeMA) framework. Six domains were considered: within-study bias, reporting bias, indirectness, imprecision, heterogeneity, and incoherence. Each comparison was assigned an overall confidence rating of high, moderate, low, or very low. Because CRP and ESR did not form connected treatment networks and were analyzed using conventional pairwise meta-analysis, their certainty was assessed using the GRADE domains of risk of bias, inconsistency, indirectness, imprecision, and publication bias. Detailed domain-level judgments are reported in **Supplementary Tables 7**-**S12**.

## 3 Results

### 3.1 Literature search results

The systematic search identified 6,237 records, including 1,493 from PubMed, 1,332 from Cochrane Library, 1,115 from Web of Science, 1,776 from Embase, 178 from CNKI, 181 from Wanfang, and 156 from Sinomed (see Appendix 1 for the detailed search results). 3,336 records were left for title and abstract screening after duplicates were eliminated. 77 articles were retrieved for full-text assessment. Ultimately, 24 randomized controlled trials met the eligibility criteria and were included in the network meta-analysis. The study selection process is presented in **Figure 1**.

### 3.2 Characteristics of included studies

A total of 24 randomized controlled trials involving 1,557 participants were included in this network meta-analysis (Ng et al., 2007; Shi and Wang, 2014; Gerbarg et al., 2015; Klare et al., 2015; Zhang et al., 2016; Cramer et al., 2017; Wu and Xie, 2017; Zhang, 2017; Ding et al., 2018; Liu, 2018; Li and Zhao, 2019; Tew et al., 2019; Hu et al., 2020; Jones et al., 2020; Seeger et al., 2020; Xie, 2020; Hu, 2021; Tao et al., 2022; Tao et al., 2023a; Tao et al., 2023b; Ding et al., 2024; Huang et al., 2025; Pan and Wen, 2025; Werner et al., 2025). Among these, 21 were two-arm trials and 3 were three-arm trials, with sample sizes ranging from 8 to 61 participants. Across the studies, exercise interventions were compared with conventional therapy (CT) or with alternative exercise regimens. Each intervention type was represented by at least two studies. The basic characteristics of the included studies are summarized in **Table 2**, with detailed information provided in **Supplementary Table 1**.

### 3.3 Risk of bias assessment

A total of 24 RCTs were included. Four studies reported adequate methods for sequence generation, including random number tables, computer-generated sequences, drawing lots, or coin tossing, and were judged to be at low risk of bias (Ng et al., 2007; Ding et al., 2018; Tao et al., 2023b; Werner et al., 2025). The remaining 20 studies stated that randomization was used but did not describe the method and were therefore rated as unclear risk. For allocation concealment, two studies used sequentially numbered opaque envelopes and were assessed as low risk (Klare et al., 2015; Cramer et al., 2017), whereas the remaining studies provided insufficient information and were rated as unclear risk.

Owing to the nature of exercise interventions, blinding of participants and personnel was generally infeasible; accordingly, all included trials were judged to be at high risk of performance bias (Ng et al., 2007; Shi and Wang, 2014; Gerbarg et al., 2015; Klare et al., 2015; Zhang et al., 2016; Cramer et al., 2017; Wu and Xie, 2017; Zhang, 2017; Ding et al., 2018; Liu, 2018; Li and Zhao, 2019; Tew et al., 2019; Hu et al., 2020; Seeger et al., 2020; Xie, 2020; Hu, 2021; Tao et al., 2022; Tao et al., 2023a; Tao et al., 2023b; Ding et al., 2024; Huang et al., 2025; Pan and Wen, 2025; Werner et al., 2025). Only one study reported blinding of outcome assessors and was therefore rated as low risk in this domain (Cramer et al., 2017), whereas the remaining studies provided insufficient information and were judged to have an unclear risk of detection bias. Regarding incomplete outcome data, nine studies adequately reported participant attrition and reasons for dropout and were rated as low risk (Ng et al., 2007; Shi and Wang, 2014; Gerbarg et al., 2015; Klare et al., 2015; Liu, 2018; Li and Zhao, 2019; Tew et al., 2019; Seeger et al., 2020; Tao et al., 2022),while the remaining studies reported complete outcome data and were also considered to be at low risk (Zhang et al., 2016; Cramer et al., 2017; Wu and Xie, 2017; Zhang, 2017; Ding et al., 2018; Hu et al., 2020; Jones et al., 2020; Xie, 2020; Hu, 2021; Tao et al., 2023a; Tao et al., 2023b; Ding et al., 2024; Huang et al., 2025; Pan and Wen, 2025; Werner et al., 2025). All included trials reported the prespecified outcomes and were therefore judged to be at low risk of selective reporting bias.

**Figure 2:**
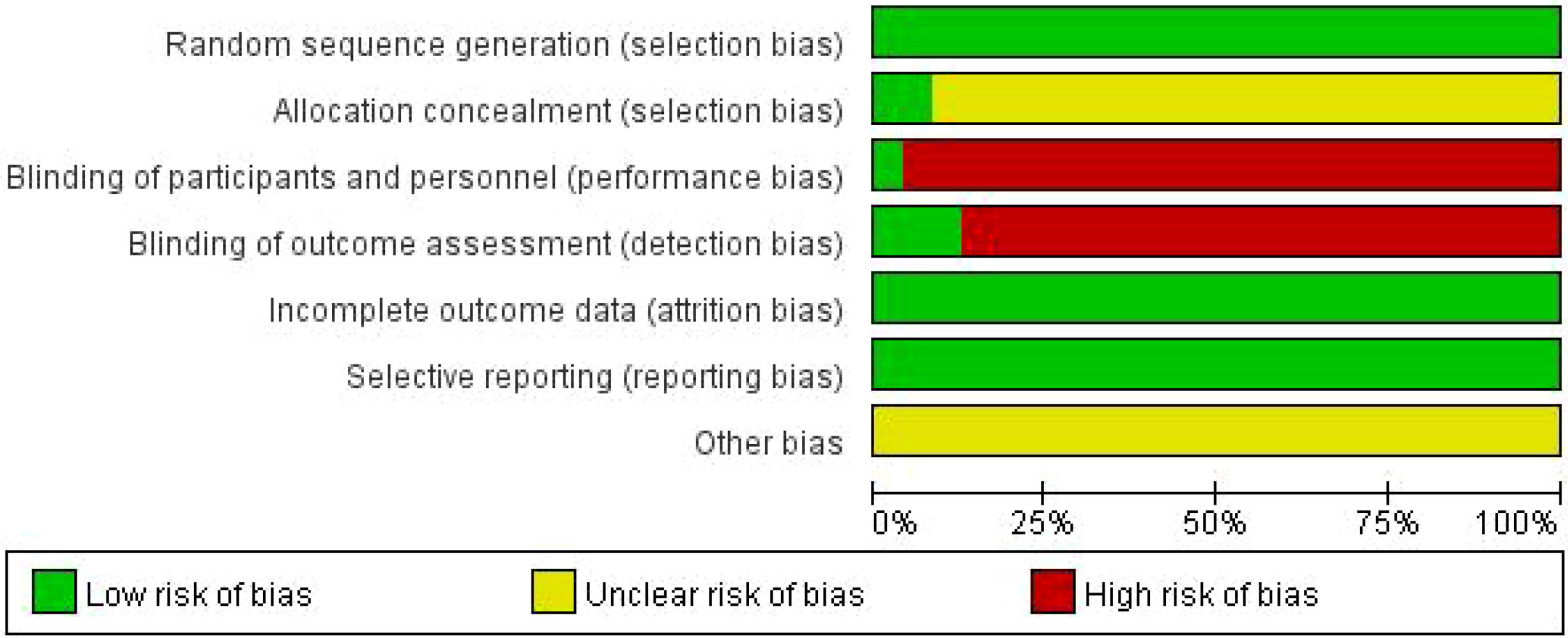
Risk of bias assessment included in the study.

In summary, the included RCTs showed risk-of-bias concerns mainly in random sequence generation, allocation concealment, and blinding, while the remaining domains were generally judged to be at low risk. The overall risk-of-bias assessment is presented in **Figure 2**, and study-level details are provided in **Supplementary Figure 1**.

### 3.4 Network meta-analysis of outcome measures

#### 3.4.1 Health-related quality of life

**Figure 3:**
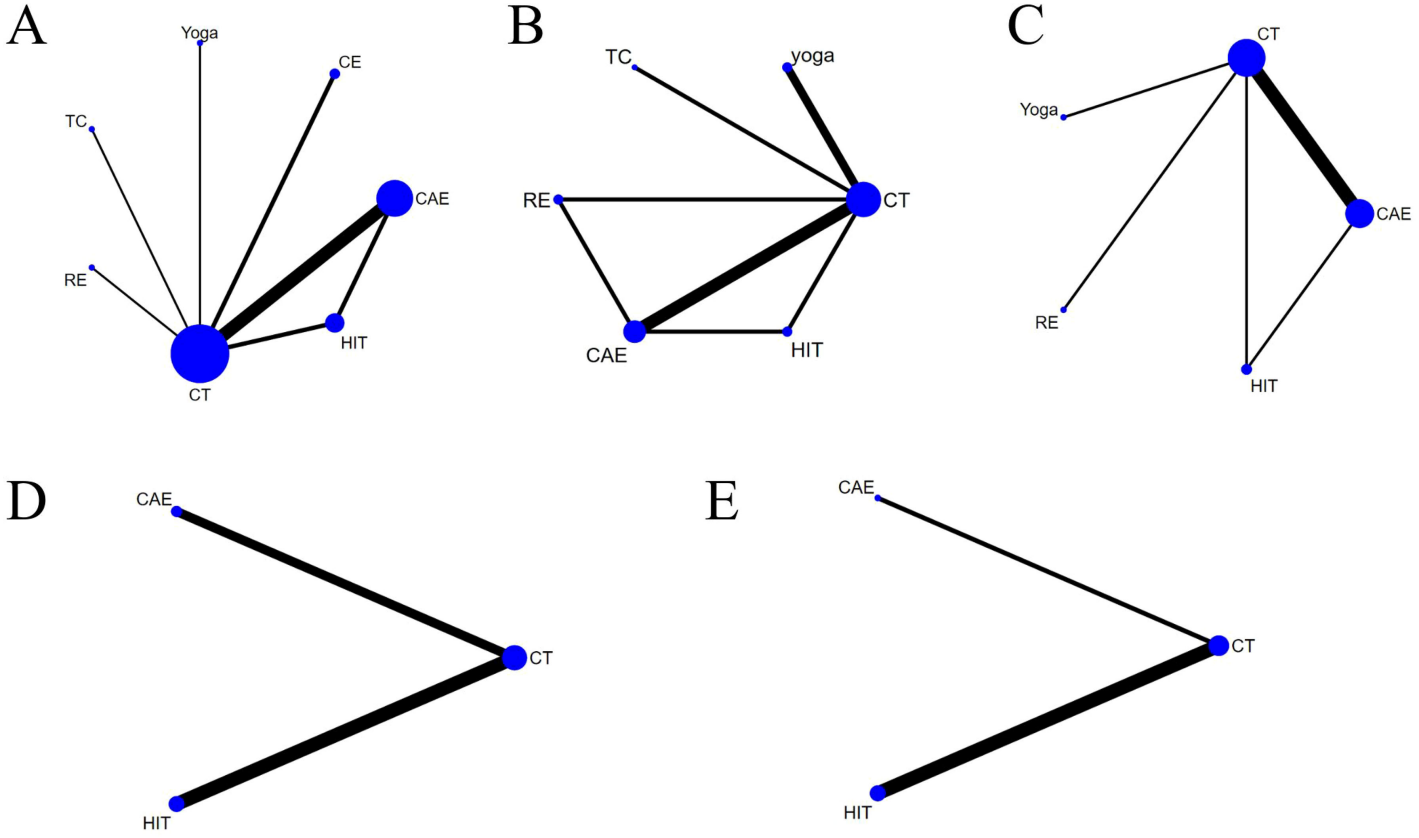
The evidence network of the included interventions across outcomes. **(A)** Quality of Life; **(B)** Disease Activity; **(C)** Fatigue Severity; **(D)** IL-6; **(E)** TNF-;i The thickness of the lines represents the number of studies, and the sizes of the nodes indicate the total sample sizes for each treatment. CT, Conventional Therapy; CE, Combined Aerobic and Resistance Exercise; CAE, Continuous Aerobic Exercise; TC, Tai Chi; RE, Resistance Exercise; HIT, High-intensity Interval Training.

A total of 15 RCTs comprising 895 participants were included in the network meta-analysis of health-related quality of life. The evidence network covered six exercise modalities, with CT serving as the common comparator. One closed loop was identified (CT-CAE-HIT), and the corresponding network plot is presented in **Figure 3A**.

Consistency between direct and indirect evidence was initially evaluated since health-related quality of life is a continuous outcome. A chi-square-based test was used to assess global inconsistency, and the results showed P > 0.05. The node-splitting approach was used to further investigate local inconsistency; all nodes had P > 0.05, suggesting no major inconsistency within the network. As a result, pool effect sizes were subjected to a consistency model.

**Figure 4:**
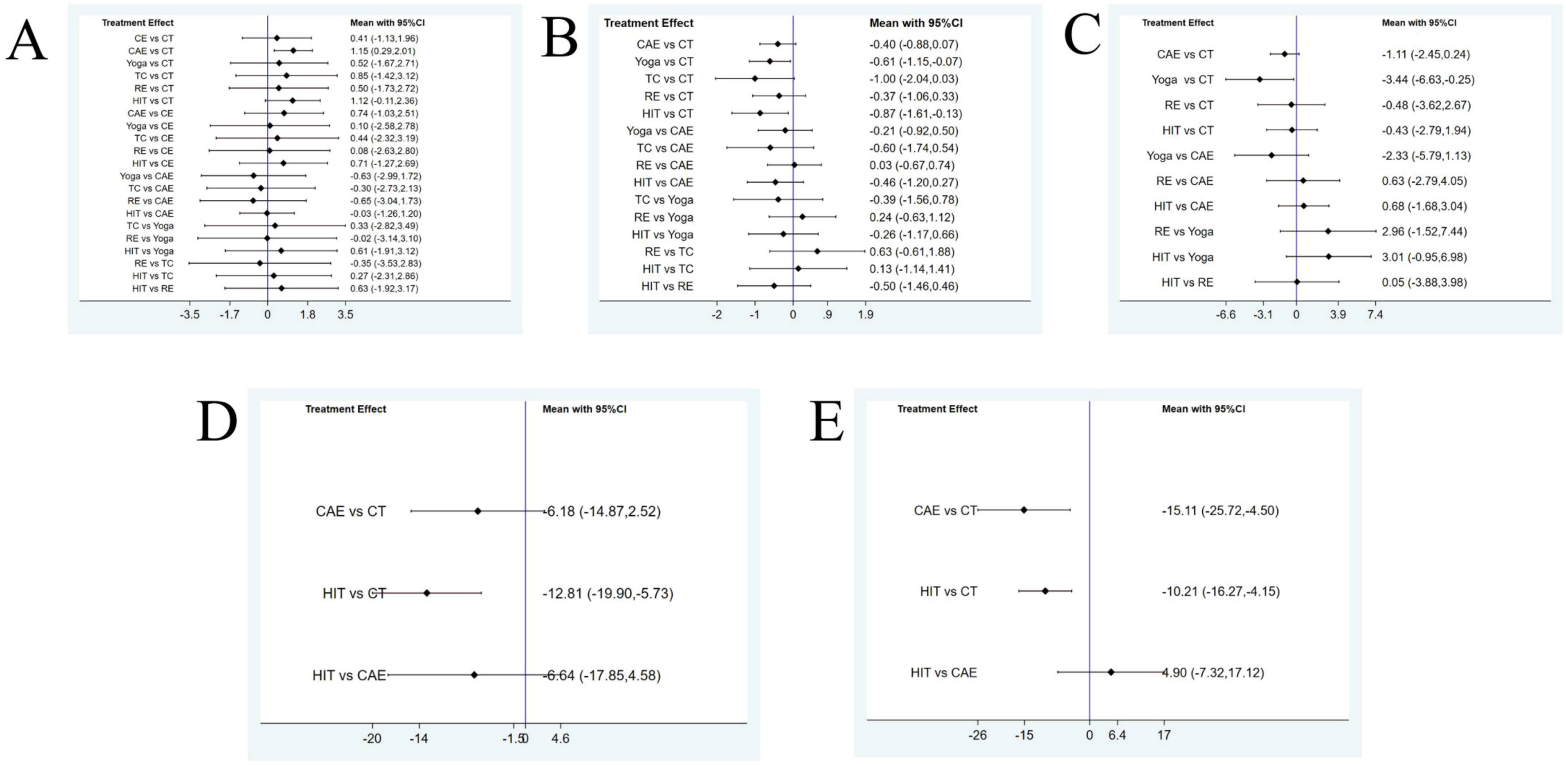
Forest plots of the network meta-analysis for the principal outcomes. **(A)** Quality of Life; **(B)** Disease Activity; **(C)** Fatigue Severity; **(D)** IL-6; **(E)** TNF-α. The figure presents pooled effect estimates with 95% confidence intervals for the principal outcomes included in the network meta-analysis. Effect estimates are expressed as mean differences or standardized mean differences, as appropriate. CT, Conventional Therapy; CE, Combined Aerobic and Resistance Exercise; CAE, Continuous Aerobic Exercise; TC, Tai Chi; RE, Resistance Exercise; HIT, High-intensity Interval Training.

Compared with CT, all exercise modalities showed effect estimates in a favorable direction for health-related quality of life, although only CAE reached statistical significance. Specifically, CAE was associated with a significant improvement in quality of life (SMD = 1.15, 95% CI [0.29, 2.01], *P* < 0.05), whereas the remaining comparisons did not reach statistical significance. **Figure 4A** displays the corresponding forest plot.

For the only closed loop in the evidence network (CT-CAE-HIT), a loop-specific inconsistency assessment was conducted. An inconsistency factor (IF) ≥ 1 typically indicates a potential trend of loop inconsistency. In this study, the IF for the loop was 2.88 (95% CI [0.00, 39.53], *P* = 0.878; **Table 3**), suggesting no statistically significant inconsistency between direct and indirect evidence, although the IF > 1 may indicate a potential trend related to heterogeneity among interventions.

**Table 3:** Loop-specific inconsistency assessment.

Outcomes	Loop	IF	95%CI	Loop-specific heterogeneity
Quality of Life	CT-CAE-HIT	2.88	(0.00,39.53)	243.887
Disease Activity	CT-CAE-HIT	0.43	(0.00,2.42)	0.248
Disease Activity	CT-CAE-RE	0.36	(0.00,2.30)	0.248
Fatigue Severity	CT-CAE-HIT	1.60	(0.00,6.69)	1.956

Conventional pairwise meta-analyses were performed for key intervention comparisons and heterogeneity was evaluated in order to test the robustness of the network meta-analysis results.Substantial heterogeneity was observed for CT versus HIT (I²= 95.2%, *P* < 0.05; **Supplementary Figure 2**) and CT versus CAE (I² = 90.8%, *P* < 0.05; **Supplementary Figure 3**). By contrast, heterogeneity for CAE versus HIT did not reach statistical significance (I² = 71.3%, *P* = 0.065; **Supplementary Figure 4**).

In the CAE subgroup, three studies from Asia (Ng et al., 2007; Liu, 2018; Tao et al., 2023a) exhibited high heterogeneity (I² = 95%, *P* < 0.05). Using a leave-one-out approach, one study (Ng et al., 2007) was identified as a major contributor to heterogeneity (MD = 3.076, 95% CI [2.493, 3.658]). After excluding this study, heterogeneity decreased from I² = 90.8% to I² = 67.9% (*P* = 0.006; **Supplementary Figure 5**), indicating that geographic region may be an important source of heterogeneity.

Sensitivity analyses were carried out by recalculating the aggregate effect size after each CAE study was successively excluded (**Supplementary Figure 6**). The results indicated minor fluctuations in effect estimates, but the main conclusion—that CAE significantly improves quality of life—remained robust. Meta-regression analyses using country/region as a moderator revealed no significant effect of region (exp = 0.24, 95% CI [0.04, 1.46], *P* > 0.05; **Supplementary Figure 7**). Additional meta-regression analyses for intervention frequency and duration indicated that neither factor had a statistically significant impact on effect size (**Supplementary Figures 8**, **S9**).

The CT–HIT comparison included only two studies (Liu, 2018; Xie, 2020), and the forest plot revealed substantial differences in effect size (Garry A: SMD = −8.00, 95% CI [−23.89, 7.89]; Zhang Y: SMD = 40.50, 95% CI [29.46, 51.54]), which were attributed to differences in intervention protocols.

**Figure 5:**
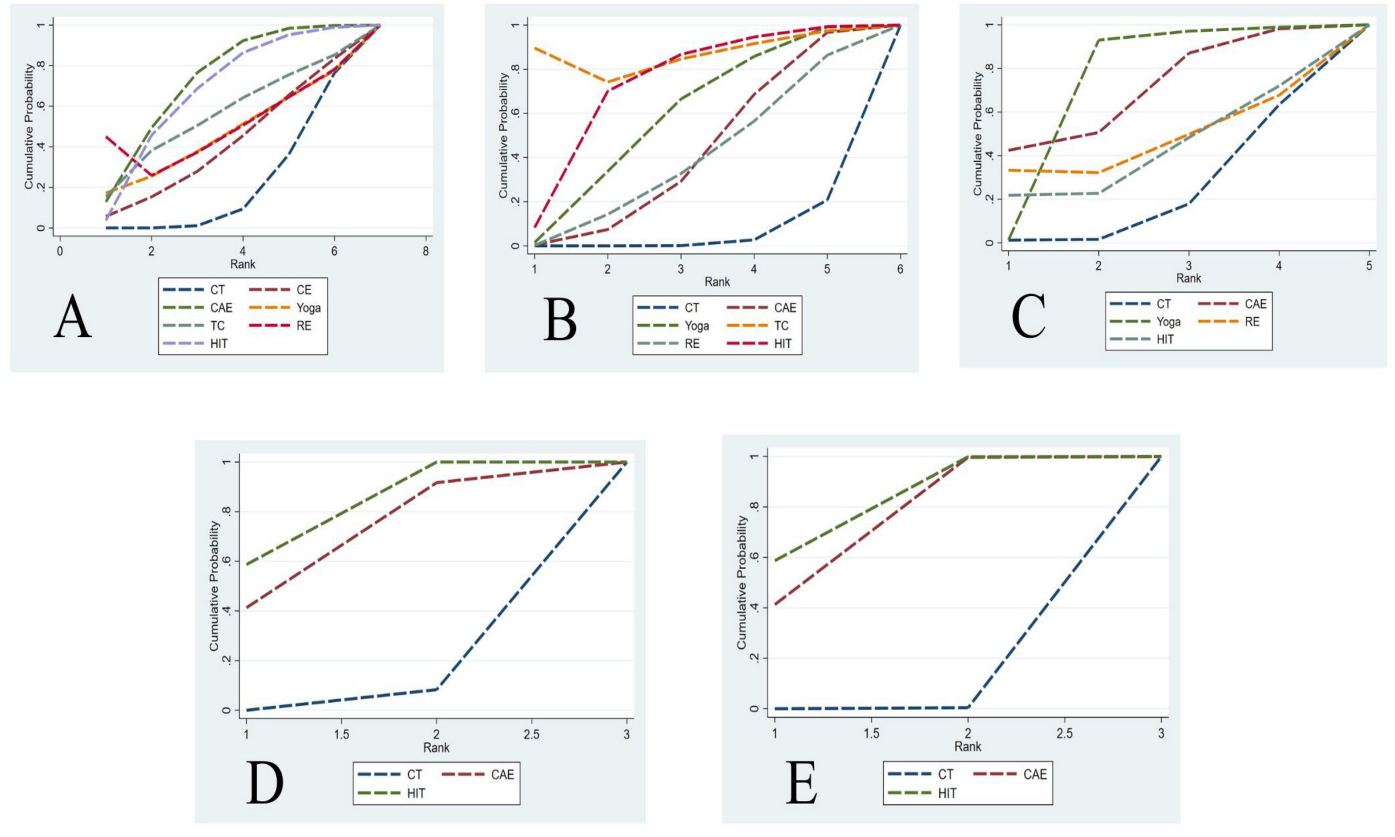
The SUCRA ranking for the principal outcomes. **(A)** Quality of Life; **(B)** Disease Activity; **(C)** Fatigue Severity; **(D)** IL-6; **(E)** TNF-α. Higher SUCRA values indicate a greater probability that an intervention ranks among the most favorable options for the corresponding outcome. CT, Conventional Therapy; CE, Combined Aerobic and Resistance Exercise; CAE, Continuous Aerobic Exercise; TC, Tai Chi; RE, Resistance Exercise; HIT, High-intensity Interval Training.

The SUCRA analysis indicated that CAE was most likely to be the best intervention for enhancing IBD patients’ quality of life. The SUCRA ranking was as follows: CAE (71.9%) > HIT > TC > yoga > RE > CE > CT (**Figure 5A**; detailed values are provided in **Table 4**). These rankings indicate relative probabilities within the evidence network and should therefore be interpreted cautiously, rather than as conclusive evidence of clinical superiority. The corresponding league table is presented in **Supplementary Table 2**.

**Table 4:** The SUCRA values of each treatment modality.

Treatment	QOL	Rank	Disease activity	Rank	IBD-F	Rank	IL-6	Rank	TNF-α	Rank
CE	40.7%	6	–	–	–	–	–	–	–	–
CAE	71.9%	1	41%	4	60.4%	2	52.4%	2	88.9%	1
Yoga	45.5%	4	59.7%	3	93%	1	–	–	–	–
TC	56.6%	3	79.4%	1	–	–	–	–	–	–
RE	44.8%	5	38%	5	39.3%	3	–	–	–	–
HIT	69.9%	2	76.7%	2	36.7%	4	93.5%	1	60.9%	2
CT	20.6%	7	5.1%	6	20.7%	5	4%	3	0.2%	3

#### 3.4.2 Disease activity

A total of 10 RCTs comprising 282 participants were included in the network meta-analysis of disease activity. The evidence network covered six exercise modalities, with CT serving as the common comparator. Two closed loops were identified (CT–RE–CAE and CT–CAE–HIT), and the corresponding network evidence plot is shown in **Figure 3B**. Because different instruments were used to assess disease activity across studies, standardized mean differences (SMDs) with 95% confidence intervals were used for quantitative synthesis.

The network meta-analysis showed that all six exercise modalities had effect estimates in a favorable direction relative to CT for disease activity. Pairwise comparisons indicated that yoga versus CT (SMD = -0.61, 95% CI [-1.15, -0.07]) and HIT versus CT (SMD = -0.87, 95% CI [-1.61, -0.13]) reached statistical significance, whereas the remaining comparisons did not (**Figure 4B**).

Consistency within the closed loops was further evaluated (**Table 3**). Loop-specific inconsistency tests showed that the CT-CAE-RE loop had an IF of 0.431 (95% CI [0.00, 2.42], *P* = 0.671), and the CT-CAE-HIT loop had an IF of 0.358 (95% CI [0.00, 2.30], *P* = 0.718), indicating no statistically significant inconsistency between direct and indirect evidence. As both IF values were below 1, heterogeneity within the loops was considered low.

In the SUCRA analysis, Tai Chi showed the highest probability of ranking as the most favorable intervention for reducing disease activity in patients with IBD. The ranking of interventions according to SUCRA values was as follows: TC > HIT (76.7%) > yoga (59.7%) > CAE > RE > CT (**Figure 5B**; detailed values in **Table 4**). The league table summarizing the efficacy ranking of interventions is provided in **Supplementary Table 3**.

#### 3.4.3 Fatigue severity

A total of 7 RCTs comprising 514 participants were included in the network meta-analysis of fatigue severity. The evidence network covered four exercise interventions. One closed loop was formed (CT-CAE-HIT), and the corresponding network evidence diagram is shown in **Figure 3C**. Accordingly, the relative effects and rankings for fatigue apply only to the modalities represented in this network.

The NMA showed that all included exercise modalities had effect estimates in a favorable direction relative to CT, although only yoga versus CT reached statistical significance (SMD = -3.44, 95% CI [-6.63, -0.25]; **Figure 4C**).

The loop formed by the interventions had an inconsistency factor (IF) of 1.60 (95% CI [0.00, 6.69], *P* = 0.537), indicating no statistically significant inconsistency between direct and indirect evidence (**Table 3C**). However, IF > 1 indicates a possible pattern of inconsistency that may be related to intervention heterogeneity.

Conventional pairwise analysis was conducted to explore heterogeneity. High heterogeneity was observed between CAE and CT (I² = 96.2%, *P* = 0.038; **Supplementary Figure 10**). For comparisons between CT and HIT, and between HIT and CAE, only one study was available for each, so heterogeneity could not be formally assessed (**Supplementary Figure 12**).

According to the SUCRA analysis, yoga had the highest probability of being the most favorable intervention for reducing fatigue severity in patients with IBD. The SUCRA-based ranking of interventions was as follows: yoga (93%) > CAE > RE > HIT > CT (**Figure 5C**; detailed values in **Table 4**). The league table summarizing the efficacy rankings of interventions is provided in **Supplementary Table 4**. Because two exercise modalities were absent and direct head-to-head evidence was sparse, this ranking should not be interpreted as establishing yoga as superior to all possible exercise approaches.

#### 3.4.4 Interleukin-6

A total of 5 RCTs comprising 441 participants were included in the network meta-analysis of IL-6, covering two exercise interventions. No closed loops were formed, and the corresponding network evidence diagram is shown in **Figure 3D**. Consequently, the analysis does not support comparisons with yoga, Tai Chi, resistance exercise, or combined aerobic and resistance exercise.

Pairwise comparisons between the exercise interventions and CT demonstrated that both interventions reduced IL-6 levels, with HIT versus CT showing a statistically significant effect (MD = −12.18, 95% CI [−19.90, −5.73], *P* < 0.05). No statistically significant differences were observed between other pairwise comparisons (*P* > 0.05; **Figure 4D**).

Based on SUCRA analysis, HIT had the highest probability of being the most favorable intervention for reducing IL-6 in patients with IBD. The ranking of interventions according to SUCRA values was as follows: HIT (93.5%) > CAE > CT (**Figure 5D**; detailed values in **Table 4**). The league table summarizing the efficacy ranking of interventions is provided in **Supplementary Table 5**. This ranking is restricted to the three-node network and should be regarded as exploratory.

#### 3.4.5 Tumor necrosis factor-α

A total of 4 RCTs involving 326 participants were included in the network meta-analysis for TNF-α, covering two exercise interventions. No closed loops were formed, and the corresponding network evidence diagram is shown in **Figure 3E**. Therefore, the findings cannot be extrapolated to the four exercise modalities absent from this outcome network.

Pairwise comparisons between the exercise interventions and CT indicated that both interventions significantly reduced TNF-α levels. Specifically, CAE versus CT (MD = −15.11, 95% CI [−25.72, −4.50]) and HIT versus CT (MD = −10.21, 95% CI [−16.27, −4.15]) reached statistical significance (*P* < 0.05). No significant differences were observed between other pairwise comparisons (*P* > 0.05; **Figure 4E**).

In the SUCRA analysis, CAE showed the highest probability of ranking as the most favorable intervention for reducing TNF-α levels in patients with IBD. The ranking of interventions according to SUCRA values was as follows: CAE (88.9%) > HIT (60.9%) > CT (**Figure 5E**; detailed values in **Table 4**). The league table summarizing the efficacy ranking of interventions is provided in **Supplementary Table 6**. This ranking is restricted to the three-node network and should not be interpreted as evidence of superiority over unrepresented modalities.

#### 3.4.6 C-reactive protein

A total of 2 RCTs comprising 84 participants were included to assess the effects of exercise interventions on CRP levels. A random-effects model was used, with the weighted mean difference (WMD) as the effect measure to compare yoga and TC.

Subgroup analysis showed that a 6-month yoga intervention (Hu, 2021) significantly reduced CRP levels (WMD = −2.78, 95% CI [−4.65, −0.91]; *P* < 0.05; **Supplementary Figure 12**). A 3-month TC intervention (Tao et al., 2023b) showed a decreasing trend (WMD = −3.84), but this did not reach statistical significance. The pooled analysis indicated an overall effect of WMD = −2.92 (95% CI [−4.66, −1.18]), suggesting that the interventions collectively produced a significant reduction in CRP levels, with low between-study heterogeneity (I² = 0.0%, *P* = 0.685).

However, because the quantitative synthesis for CRP was restricted to a very limited evidence base of only two trials, these findings are accompanied by wider confidence intervals and substantial statistical imprecision. These estimates must be interpreted with extreme caution as preliminary, exploratory trends rather than robust clinical conclusions.

#### 3.4.7 Erythrocyte sedimentation rate

A total of 2 randomized controlled trials involving 84 participants were included. A random-effects meta-analysis was performed using the weighted mean difference (WMD) as the effect measure to assess the effects of Yoga and Tai Chi on ESR levels.

Subgroup analysis showed that a 3-month Tai Chi intervention (Tao et al., 2023b) significantly reduced ESR levels (WMD = -9.439, 95% CI [-15.68, -3.18]), as shown in **Supplementary Figure 13**. The remaining subgroup did not reach statistical significance. The pooled analysis showed an overall significant effect of Tai Chi and Yoga on reducing ESR levels (WMD = -6.87, 95% CI [-10.91, -2.83]), with low between-study heterogeneity (I² = 9.7%, P = 0.293).

Although the subgroup results suggested a larger effect for the 3-month intervention than for the 6-month intervention, each subgroup included only one study. In addition, the intervention protocols differed between the two studies. Therefore, no definitive conclusion can be drawn regarding the relationship between intervention duration and effect size. Further homogeneous studies are needed to validate these findings. The associated broad statistical uncertainty underscores the need for further homogeneous, adequately powered clinical trials to validate whether these inflammatory declines are truly driven by the specific mind-body exercise modalities.

### 3.5 Subgroup analysis

High heterogeneity was observed for the outcomes of health-related quality of life and fatigue severity. Extremely high overall heterogeneity was seen in the network meta-analysis of CAE for enhancing quality of life in IBD patients (I²= 90.8%, *P* < 0.001). To explore potential sources of heterogeneity, subgroup analyses were conducted. Seven studies assessed the effects of CAE on quality of life, indicating that exercise frequency, intervention duration, and geographic region significantly influenced the outcomes. These analyses were exploratory and aimed to identify potential patterns, rather than to establish definitive effect modifiers.

Using conventional meta-analysis, comparisons by geographic region revealed substantial differences in effect size. Studies conducted in Asia (Ng et al., 2007; Liu, 2018; Tao et al., 2023a) showed the largest pooled effect (SMD = 1.99, 95% CI [0.24–3.73]), which was higher than studies from Europe (Klare et al., 2015; Xie, 2020) (SMD = 0.48) and North America (Gerbarg et al., 2015)(SMD = 0.88). These differences may reflect variations in baseline patient characteristics (e.g., initial quality of life, disease perception), sociocultural context, and healthcare follow-up practices. Notably, the Asian subgroup exhibited extremely high heterogeneity (I² = 95.0%, *P* < 0.001), with all studies from China, suggesting that differences in intervention implementation within China may be a major source of heterogeneity. In contrast, the European subgroup showed more consistent and moderate effects across three studies (I² < 50%), indicating relatively stable but non-significant effects of CAE under European healthcare conditions (**Supplementary Figure 14**).

Further analysis within China revealed significant differences in effect sizes across provinces, with overall heterogeneity remaining high (I² = 95.0%, *P* < 0.001). Specifically, two studies conducted in Jiangsu Province (Ng et al., 2007; Tao et al., 2023a) showed very large effect differences and heterogeneity as high as 97.4%, likely contributing substantially to the overall heterogeneity (**Supplementary Figure 15**). These variations may be due to differences in intervention protocols (exercise supervision intensity, adherence support), healthcare resources, baseline quality of life, regional culture, and follow-up systems. Future research should carefully consider regional characteristics when developing exercise recommendations.

Subgroup analyses based on weekly exercise frequency suggested a potential dose-response relationship (**Supplementary Figure 16**). Interventions delivered four times per week (Ng et al., 2007) yielded the largest effect size (SMD = 3.08), which may suggest that this frequency represents a relatively favorable aerobic exercise prescription for patients with IBD. Interventions conducted three times per week (Klare et al., 2015; Liu, 2018; Xie, 2020; Tao et al., 2023a; Werner et al., 2025), the most common frequency, yielded a moderate pooled effect (SMD = 0.79), although within-group heterogeneity remained high (I² = 74.1%), indicating that variations in intensity, session duration, and individual differences may contribute to outcome fluctuations. High-frequency interventions performed seven times per week (Gerbarg et al., 2015) (SMD = 0.87) did not outperform the four-times-per-week group and were even lower than some three-times-per-week studies, suggesting that exceeding a certain frequency may reduce perceived benefits due to fatigue, adherence challenges, or overtraining risk (**Supplementary Figure 17**). These findings suggest that four sessions per week may balance exercise dose, adherence, and clinical efficacy.

Further analyses examined the impact of intervention duration. Three-month interventions (Ng et al., 2007; Liu, 2018; Xie, 2020; Werner et al., 2025) produced the largest pooled effect size (SMD = 1.58), but heterogeneity was extremely high (I² = 93.3%), indicating that three months may be a critical period for the full manifestation of intervention effects. During this period, differences in program quality (supervision intensity, individualized adjustments) may be amplified, resulting in outcome variability. Interventions of 2.5 months (Klare et al., 2015; Tao et al., 2023a) demonstrated good homogeneity (I² = 0%) but a smaller effect size (SMD = 0.49), suggesting insufficient duration may limit cumulative benefits. A single 7-month study (Gerbarg et al., 2015) showed a reduced effect size (SMD = 0.87) compared with the three-month interventions (**Supplementary Figure 17**). These findings suggest that the impact of continuous aerobic exercise on quality of life may vary according to several factors, including geographic setting as well as training frequency and program duration. Taken together, the current evidence suggests that an intervention delivered four times weekly for three months may represent a potentially favorable prescription pattern for quality-of-life improvement in IBD and may help inform the design of future randomized controlled trials. However, these findings should be viewed as hypothesis-generating rather than conclusive as they came from exploratory subgroup analyses and were not supported by meta-regression.

Subgroup analyses were also conducted to evaluate CAE’s effect on fatigue severity. When stratified by country, pooled effects from Asian studies were statistically significant (SMD = −1.62, 95% CI [−3.04, −0.20], *P* = 0.025), whereas a UK study showed no significant effect (*P* = 0.468; **Supplementary Figure 19**). However, unexplained heterogeneity remained high within the Asian subgroup (I² = 96.2%), suggesting that national differences only partially explain observed variability. Subgroup analysis by intervention duration indicated that heterogeneity within the three-month subgroup remained very high (I² = 97.0%; **Supplementary Figure 18**). Leave-one-out analyses did not significantly reduce heterogeneity (**Supplementary Figure 20**). Meta-regression analyses of intervention duration and country (**Supplementary Figures 21**, **S22**) suggested that the observed variability may result from complex interactions among multiple study-level factors below the national level.

**Figure 6:**
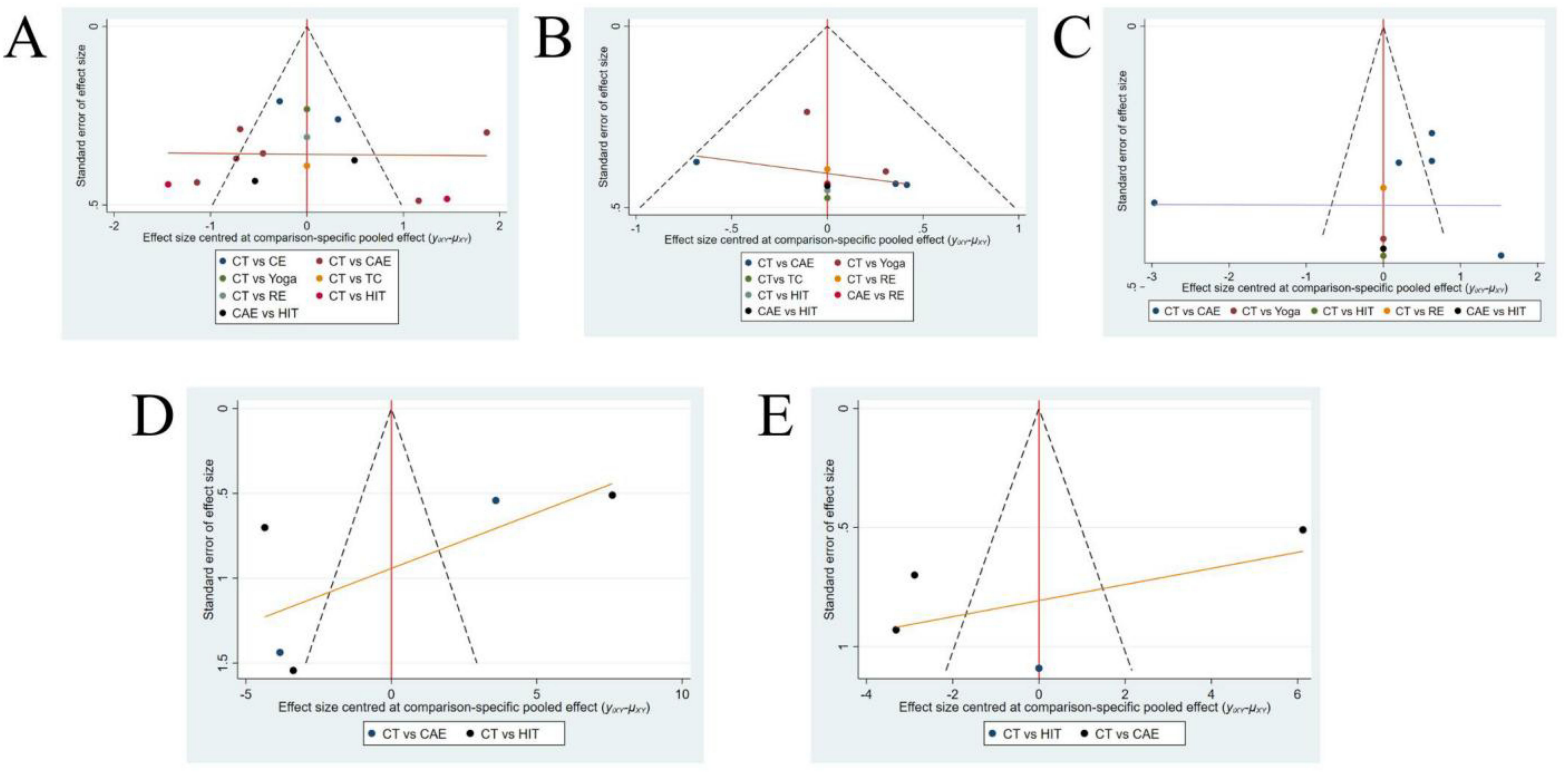
Funnel plot of each intervention. **(A)** Quality of Life; **(B)** Disease Activity; **(C)** Fatigue Severity; **(D)** IL-6; **(E)** TNF-α.CT, Conventional Therapy; CE, Combined Aerobic and Resistance Exercise; CAE, Continuous Aerobic Exercise; TC, Tai Chi; RE, Resistance Exercise; HIT, High-intensity Interval Training.

Detailed examination of intervention protocols and study designs in the Asian studies suggested several potential sources of heterogeneity. Intervention protocols varied substantially; for example, TaoMQ2023 (Tao et al., 2022) implemented different supervision conditions, including fully in-hospital supervision versus home-based remote supervision. Study design rigor varied, including whether interventions were evidence-based, incorporated expert consultation, and emphasized patient adherence strategies. Outcome assessment differed across studies: TaoMQ2023 evaluated fatigue (MFI-20) and included lower-limb isokinetic strength testing, TaoMQ2022 (Ng et al., 2007) assessed both quality of life and inflammatory markers, and Ding WQ2024 (Shi and Wang, 2014) primarily used the IBDQ to assess quality of life. Although fatigue severity was the primary outcome in all studies, differences in measurement scales and study design focus on physiological versus psychological dimensions may have introduced subjective variation in fatigue assessment. Consequently, even within the same country and intervention duration, substantial variability in study effects may occur due to these multiple factors. Publication bias and small-study effects were visually assessed using comparison-adjusted funnel plots for the five network outcomes, including quality of life, disease activity, fatigue severity, IL-6, and TNF-α (**Figures 6A–E**). Overall, no obvious or consistent asymmetry was visually observed across the plots. However, this finding should be interpreted cautiously because several outcome networks, particularly those for fatigue severity, IL-6, and TNF-α, were based on a limited number of studies and sparse direct comparisons. Therefore, the funnel plots provided no clear visual evidence of substantial publication bias, although small-study effects or reporting bias could not be fully excluded.

### 3.6 Adverse reactions

A total of 9 studies reported adverse reactions (ARs), whereas 15 studies reported no ARs. Overall, 36 exercise-related non-serious ARs were documented (**Table 5**), including muscle soreness, gastrointestinal symptoms, abdominal pain, diarrhea, dizziness, and headache. Four serious ARs were reported in studies by Garry A (Seeger et al., 2020) and H. Cramer (Cramer et al., 2017), including chest infection, newly diagnosed colorectal cancer, and acute disease flare; these events were considered unrelated to the exercise interventions. No other studies reported serious ARs.

**Table 5:** Adverse events associated with different treatment.

Treatment	Abdominal symptoms	Headache	Xerostomia	Exercise-related symptoms	Others
CE	–	–	–	–	–
CAE	8	2	1	5	2
Yoga	3	–	–	6	–
TC	–	–	–	–	–
RE	1	2	–	–	–
HIT	5	3	–	6	2
CT	–	–	–	–	–

### 3.7 Certainty of evidence

CINeMA assessments indicated that confidence in the network estimates ranged from moderate to very low. For health-related quality of life, confidence was moderate for several comparisons with CT (CAE, resistance exercise, and HIT), low for CT versus combined exercise and CAE versus HIT, and very low for most indirect comparisons. For disease activity and fatigue severity, confidence was predominantly low or very low, with moderate confidence limited to selected comparisons with CT. Confidence in the IL-6 and TNF-α networks was low or very low. Conventional GRADE assessment rated the evidence for CRP and ESR as very low certainty. Domain-level judgments and comparison-specific ratings are provided in **Supplementary Tables 7**-**S12**.

## 4 Discussion

### 4.1 Summary and interpretation of the study

This network meta-analysis evaluated up to six exercise modalities, with CT as the common comparator, across quality of life, disease activity, fatigue, IL-6, TNF-α, CRP, and ESR in patients with IBD. Overall, exercise-based interventions may benefit several patient-reported and clinical outcomes, although effect magnitude, consistency, network coverage, and confidence varied substantially across endpoints.

With regard to quality of life, continuous aerobic exercise showed the most favorable overall pattern and ranked highest in the SUCRA analysis. Subgroup findings further suggested that an intervention pattern involving four sessions per week over three months may represent a relatively preferable prescription for quality-of-life improvement in this population and may provide a useful reference for future randomized controlled trials, although this observation should be regarded as exploratory. For disease activity, Tai Chi showed the most favorable ranking profile, while yoga and HIT were the interventions that reached statistical significance versus CT. For fatigue severity, yoga appeared to be the most favorable intervention on the basis of both effect estimates and ranking probability.

Regarding inflammatory biomarkers, HIT was associated with reduced IL-6 levels, whereas both continuous aerobic exercise and HIT showed favorable effects on TNF-α. In addition, pooled analyses provided preliminary evidence that yoga and Tai Chi may contribute to lower CRP and ESR levels. Taken together, these findings suggest that different exercise modalities may confer distinct benefits across outcome domains in IBD.

Importantly, intervention coverage was outcome-dependent. All six exercise modalities were represented only in the quality-of-life network; the disease-activity network lacked combined aerobic and resistance exercise, the fatigue network lacked Tai Chi and combined exercise, and the IL-6 and TNF-α networks included only continuous aerobic exercise and high-intensity interval training. Thus, the SUCRA rankings answer a narrower question—how the available modalities compare within each observed network—rather than which exercise is best among all plausible options. This limitation, together with predominantly low or very-low confidence ratings, requires conservative interpretation and precludes recommendations for or against modalities that were not represented.

### 4.2 Comparison with other studies

Compared with previous reviews and meta-analyses, this study extends the current evidence base by directly comparing multiple exercise modalities within a unified network framework. It also evaluates a broader range of outcomes, including quality of life, disease activity, fatigue, and inflammatory biomarkers. For instance, a 2025 review (Al-Beltagi et al., 2025) reported that combined yoga and TC interventions may modulate the gut–brain axis, reduce cortisol levels, and improve overall physiological health, indicating a pathophysiological connection between mental health and IBD. The review further suggested that regular moderate-intensity exercise could alleviate inflammation, enhance intestinal mucosal barrier integrity, and improve quality of life, which aligns with the present study’s findings regarding improvements in quality of life, fatigue severity, and inflammatory markers, while also providing additional mechanistic insights.

A 2022 narrative review (Raman et al., 2022), together with several case-control studies, reported that exercise interventions were associated with improvements in quality of life, fatigue severity, and anxiety or depression among patients with IBD. These findings are broadly consistent with our results. Conversely, a 2022 meta-analysis (Jones et al., 2022) reported no significant effect of structured exercise on health-related quality of life, which appears to contradict the present findings. This discrepancy may be attributable to the limited number of included studies, low certainty of evidence, and heterogeneity in exercise interventions. Notably, the authors noted that sensitivity analyses excluding one study revealed improvements in quality of life with a substantial reduction in heterogeneity. In meta-analyses that did not include quality-of-life outcomes, all but one study reported clinically meaningful improvements following exercise interventions.

Regarding IBD-related comorbidities, a systematic review (Protano et al., 2026) indicated that physical activity may confer protective effects on musculoskeletal and cardiovascular health in patients with IBD. However, the underlying biological mechanisms remain unclear, highlighting the need for further research into the impact of exercise on IBD-associated complications.

With respect to fatigue severity, most studies are consistent with the present findings, although the magnitude of improvement varied depending on exercise type and duration. This suggests that exercise can alleviate fatigue symptoms. However, reporting of intervention duration and frequency was incomplete in some studies. Future research should therefore quantitatively evaluate the duration, frequency, and intensity of specific exercise interventions to better inform personalized exercise prescriptions for patients with IBD.

### 4.3 Potential mechanisms of exercise in alleviating IBD symptoms

While the randomized controlled trials included in this network meta-analysis primarily focused on clinical endpoints and did not directly evaluate real-time molecular pathways, several potential physiological mechanisms can be hypothesized to contextualize these exercise-induced adaptations.

#### 4.3.1 Continuous aerobic exercise

Continuous aerobic exercise may potentially influence IBD-related outcomes through several interconnected, hypothetical pathways, including inflammatory regulation, stress adaptation, barrier maintenance, and modulation of the gut microbiota. Previous basic science literatures suggest that long-term aerobic physical stress might modulate genes involved in stress adaptation, thereby contributing to mucosal homeostasis (Yoon et al., 2024). Aerobic exercise may also influence barrier-associated molecules, including MUC2, occludin, and claudin-related proteins. It may further exert anti-inflammatory effects by altering the diversity and composition of the gut microbiota (Li et al., 2024; Min et al., 2024).

#### 4.3.2 Yoga

Yoga, as an ancient traditional exercise originating from India, exerts beneficial effects on both physiological and psychological mechanisms through postures, breathing exercises, and meditation. Modulation of the gut-brain axis is one of the main ways that yoga may affect IBD. Previous studies have suggested that yoga can affect the gut–brain axis by regulating the autonomic nervous system (ANS), particularly through vagus nerve stimulation to enhance parasympathetic nervous system (PNS) activity, thereby restoring homeostasis (Streeter et al., 2012). Additionally, yoga practice can effectively regulate the hypothalamic–pituitary–adrenal (HPA) axis, reducing cortisol levels and alleviating stress and inflammatory responses (Pascoe and Bauer, 2015). Psychologically, yoga may improve anxiety and depressive symptoms by enhancing GABAergic neurotransmission and modulating neuroendocrine function (Streeter et al., 2012).

#### 4.3.3 Combined aerobic and resistance training

Aerobic and resistance training may improve body composition and muscle strength. In renal transplant recipients, combined training has been shown to reduce body fat percentage, increase lean body mass and muscle strength, and improve renal function (Pascoe and Bauer, 2015). These findings suggest that combined training may improve not only body composition and muscular strength but also metabolic and cardiovascular health (Lima et al., 2021).

Furthermore, another study demonstrated that voluntary exercise can protect mice from ulcerative colitis by upregulating PPAR-γ activity in the colon (Liu et al., 2015). These findings suggest that combined training may confer protective effects in IBD by modulating inflammatory responses and improving gut microbiota composition. Combined training has also shown potential benefits for cardiovascular function. Specifically, combined exercise can reduce arterial stiffness and improve glycemic control and cardiovascular risk factors in patients with diabetes (Manojlović et al., 2021; Ma et al., 2025).

Overall, these results further support the potential application of combined aerobic and resistance training in the management of chronic diseases, including IBD.

#### 4.3.4 Resistance exercise

Resistance training may exert beneficial effects by improving intestinal barrier function. Impairment of the gut barrier is considered a key factor in the pathogenesis of ulcerative colitis. Studies have shown that after eight weeks of resistance training, participants showed a significant reduction in serum lipopolysaccharide-binding protein (LBP) levels, whereas levels tended to increase in the control group. This suggests that resistance training may enhance gut barrier integrity by reducing intestinal permeability (Dow et al., 2025).

Numerous studies have also proven the modulatory effects of resistance exercise on the immune system. For example, one study reported that resistance training promoted M2 macrophage polarization and reduced TNF-α and nuclear factor-κB (NF-κB) mRNA levels, thereby attenuating skeletal muscle inflammation in aged mice (Cao et al., 2025).

#### 4.3.5 Tai Chi

Tai Chi, a traditional Chinese exercise, has been associated with multiple health benefits. Tai Chi improves cardiovascular function and mental health (Mori et al., 2020). In addition, Tai Chi has been demonstrated to alleviate depressive symptoms and improve serum lipid profiles, which is particularly relevant for patients with chronic diseases.

From a neurophysiological perspective, Tai Chi may positively influence cognitive function and mental health by modulating brain structure and functional connectivity. A neuroimaging study (Yu et al., 2018) revealed that Tai Chi can alter brain morphology and functional networks, suggesting that improvements in functional connectivity and regional brain activity may underlie the cognitive and psychological benefits of Tai Chi. Such neuromodulatory mechanisms may represent one of the potential pathways by which Tai Chi alleviates symptoms in patients with IBD.

#### 4.3.6 High-intensity interval training

High-intensity interval training (HIT), as an effective exercise modality, exerts multisystem effects involving skeletal muscle, the cardiovascular system, and the immune system. Studies have demonstrated that HIT can induce both local and systemic modulation of molecular sensors and mediators through metabolic, mechanical, neural, and hormonal stimuli, thereby promoting adaptive changes in tissues and organs (Wahl et al., 2022).

In skeletal muscle, the high metabolic perturbations induced by HIT serve as a primary stimulus for muscular adaptation. Within the cardiovascular system, HIT triggers hemodynamic stress and modulates calcium handling, providing key stimuli for structural and functional adaptations of the heart and vasculature. Moreover, HIT influences immune function by altering signal transduction pathways, leading to both local and systemic inflammatory or anti-inflammatory responses (Goldberg et al., 2021).

In the context of IBD, the therapeutic effects of HIT may be partially mediated through modulation of the gut microbiota (Peng et al., 2024). Additionally, HIT has demonstrated potential in improving functional capacity in patients with chronic diseases, highlighting its broader applicability in disease management.

### 4.4 Limitations

This network meta-analysis summarized the comparative effects of various exercise interventions on quality of life in patients with IBD, providing guidance for clinical exercise prescriptions. However, several limitations should be acknowledged. First, apart from objective circulating biomarkers, the primary patient-reported outcomes—specifically health-related quality of life and fatigue severity—exhibited substantial statistical heterogeneity, with I^2^>90% in several major analyses. This high variability is largely driven by inherent clinical and methodological diversities across the trials, including variations in exercise supervision protocols (in-hospital versus home-based), individual patient baseline statuses (clinical remission versus mildly active phases), and individualized program adherence support. Furthermore, owing to the pragmatic nature of structured exercise training, double-blinding of participants and personnel was virtually unfeasible across the included randomized controlled trials. Given that quality of life and fatigue severity are inherently subjective, patient-reported metrics, the absence of strict blinding introduces potential performance and detection biases, such as participant expectation effects, which might overestimate the true therapeutic impacts. Consequently, these pooled estimates and relative ranking profiles must be interpreted with caution when expanding to general clinical practice, and future multi-arm trials with standardized blinding of independent outcome assessors are highly warranted.

Second, the scope of the literature review was restricted. Although studies published in English and Chinese were included, literature in other languages was not considered. Additionally, some outcome measures (IL-6, TNF-α, ESR, CRP) were represented by only one or two studies, which generally had small sample sizes. This limited representation may introduce bias. Specifically, the small sample sizes in these studies were often insufficient to achieve optimal statistical power, hindering comprehensive characterization of overall effects and potentially leading to biased conclusions. In addition, intervention coverage differed by outcome: all six exercise modalities were represented only for quality of life, whereas disease activity, fatigue, IL-6, and TNF-α were informed by progressively smaller subsets of modalities. The absence of a modality from an outcome network indicates a lack of eligible comparative evidence for that endpoint, not evidence of no effect.

Third, formal CINeMA and GRADE assessments showed that confidence in most comparisons was low or very low, particularly for sparse networks and indirect comparisons. These ratings reflect concerns related mainly to imprecision, heterogeneity, incoherence, and limited direct evidence. Accordingly, the relative efficacy hierarchies and ranking probabilities should be treated as hypothesis-generating rather than as definitive evidence of clinical superiority.

### 4.5 Implications for practice

The NMA provides a more structured comparison of exercise interventions for improving quality of life in patients with IBD. The findings may inform exercise-based supportive care and rehabilitation planning, particularly for patients in remission. However, exercise prescriptions should be individualized according to disease activity, symptom tolerance, physical capacity, and clinical context. Any modality-specific recommendation should be confined to the outcomes and interventions actually represented in the corresponding network, and no inference should be made about balance training, progressive muscle relaxation, or other unrepresented approaches.

## 5 Conclusion

In summary, the NMA systematically examined the associations of different exercise interventions with quality of life, fatigue severity, disease activity, and inflammatory biomarkers in patients with inflammatory bowel disease. The available evidence suggests that exercise-based interventions are generally associated with improvement across several patient-reported and clinical outcomes, although the magnitude and consistency of these effects appear to differ across exercise modalities. Because intervention coverage was incomplete for all outcomes except quality of life, comparisons across modalities should be interpreted within the boundaries of each outcome-specific evidence network.

Among the evaluated interventions, continuous aerobic exercise showed a comparatively favorable profile for improving quality of life. Subgroup analyses further suggested that exercise performed four times per week for three months may be associated with greater improvement in quality of life in patients with IBD. This finding may inform the design of future RCTs, but should be interpreted as exploratory. Tai Chi showed a comparatively favorable ranking pattern for reducing disease activity, while yoga appeared to be more advantageous for alleviating fatigue severity. Different exercise modalities were also associated with distinct patterns of change in inflammatory biomarkers, suggesting that their potential benefits may involve different physiological pathways.

These findings should nevertheless be interpreted with caution because the current evidence is characterized by methodological heterogeneity, variation in intervention design, and a limited number of direct comparative studies. Among the evaluated interventions, continuous aerobic exercise showed a comparatively favorable mathematical profile for improving quality of life, and Tai Chi ranked highest in probability for reducing disease activity. However, these rankings derive strictly from relative probabilities within the available network and do not establish definitive clinical superiority, especially considering the variations in the volume of supporting evidence across modalities. Future studies should prioritize adequately powered RCTs with standardized intervention protocols and outcome assessment methods. This would help clarify the comparative effectiveness of specific exercise modalities in patients with IBD.

## Data Availability

The original contributions presented in the study are included in the article/**Supplementary Material**. Further inquiries can be directed to the corresponding author.
