# Supplementary material for: Effects of exercise therapy on quality of life in patients with inflammatory bowel disease: a network meta-analysis
Source: Front Physiol. 2026 Jul 22;17:1894474. doi: 10.3389/fphys.2026.1894474 (PMC13436954; doi:10.3389/fphys.2026.1894474)
Supplement: Supplementary file 1 [file SupplementaryFile1.docx]

**SUPPLEMENTARY MATERIAL**

**Table of Contents**

**Supplementary Methods 1** – Database search strategy.

**Supplementary Figure S1** – Risk of bias assessment included in the study.

**Supplementary** **Figure S2** – Comparative Analysis of HIT versus Conventional Therapy in the Quality of Life Subgroup

**Supplementary Figure S3** – Comparative Analysis of CAE versus Conventional Therapy in the Quality of Life Subgroup

**Supplementary Figure S4** – Comparative Analysis of CAE versus HIT in the Quality of Life Subgroup

**Supplementary Figure S5** – Heterogeneity in the CAE subgroup analysis of quality of life after exclusion of Chinese studies

**Supplementary Figure S6** – Sensitivity analysis of CAE for improving quality of life

**Supplementary Figure S7** –Subgroup analysis of CAE for quality of life and meta-regression analysis by different country

**Supplementary Figure S8** –Subgroup analysis of CAE for quality of life and meta-regression analysis by different frequencies

**Supplementary Figure S9**–Subgroup analysis of CAE for quality of life and meta-regression analysis by different duration time

**Supplementary Figure S10** –Subgroup analysis of the effect of sustained aerobic exercise on fatigue level

**Supplementary Figure S11** –Subgroup analysis of the effects of different exercise modalities on fatigue level

**Supplementary Figure S12** –Subgroup analysis of the effects of different exercise modalities on C-reactive protein (CRP)

**Supplementary Figure S13** –Subgroup analysis of the effects of different exercise modalities on ESR

**Supplementary Figure S14** –Subgroup analysis of the effect of CAE across different countries on quality of life

**Supplementary Figure S15** –Subgroup analysis of the effect of different city of CAE on quality of life

**Supplementary Figure S16**–Subgroup analysis of the effect of different intervention frequencies of CAE on quality of life

**Supplementary Figure S17** –Subgroup analysis of the effect of different intervention timings of CAE on quality of life

**Supplementary Figure S18**–Subgroup analysis of the effect of different intervention timings of CAE on fatigue severity

**Supplementary Figure S19** –	Subgroup analysis of the effect of CAE across different countries on fatigue severity

**Supplementary Figure S20**–Sensitivity analysis of CAE for improving fatigue severity

**Supplementary Figure S21**–Subgroup analysis of CAE for fatigue severity and meta-regression analysis by different country

**Supplementary Figure S22**–Subgroup analysis of CAE for fatigue severity and meta-regression analysis by different time

**Supplementary Table 1 –** Basic information of all the papers

**Supplementary Table 2 –** Results of network Meta-Analysis(IBDQ)

**Supplementary Table 3** – Results of network Meta-Analysis(CDAI)

**Supplementary Table 4** – Results of network Meta-Analysis(IBD-F)

**Supplementary Table 5** – Results of network Meta-Analysis(IL-6)

**Supplementary Table 6** – Results of network Meta-Analysis(TNF-α)

**Supplementary Table 7 –** CINeMA confidence assessment for IBDQ

**Supplementary Table 8 –** CINeMA confidence assessment for Disease

Activity

**Supplementary Table 9 –** CINeMA confidence assessment for IBD-F

**Supplementary Table 10 –** CINeMA confidence assessment for IL-6

**Supplementary Table 11 –** CINeMA confidence assessment for TNF-α

**Supplementary Table 12 –** Conventional GRADE assessment for CRP and ESR

**Supplementary Methods 1**

**Pubmed**

((((((((((((((((((((((((Inflammatory Bowel Diseases[MeSH Terms]) OR (inflammatory bowel disease[Title/Abstract])) OR (IBD[Title/Abstract])) OR (Colitis Ulcerative[MeSH Terms])) OR (ulcerative colitis[Title/Abstract])) OR (UC[Title/Abstract])) OR (Crohn Disease[MeSH Terms])) OR (Crohn's disease[Title/Abstract])) OR (Crohn disease[Title/Abstract])) OR (regional enteritis[Title/Abstract])) OR (Crohn's Enteritis[Title/Abstract])) OR (Regional Enteritis[Title/Abstract])) OR (Ileocolitis[Title/Abstract])) OR (Ileitis, Terminal[Title/Abstract])) OR (Terminal Ileitis[Title/Abstract])) OR (Ileitis, Regional[Title/Abstract])) OR (Regional Ileitides[Title/Abstract])) OR (Regional Ileitis[Title/Abstract])) OR (Enteritis, Granulomatous[Title/Abstract])) OR (Granulomatous Enteritis[Title/Abstract])) OR (Enteritis, Regional[Title/Abstract])) OR (Colitis, Granulomatous[Title/Abstract])) OR (Granulomatous Colitis[Title/Abstract])) AND (((((((((((("Exercise Therapy"[Mesh]) OR ("Rehabilitation Exercise"[Title/Abstract])) OR ("Exercise, Rehabilitation"[Title/Abstract])) OR ("Exercises, Rehabilitation"[Title/Abstract])) OR ("Rehabilitation Exercises"[Title/Abstract])) OR ("Therapy, Exercise"[Title/Abstract])) OR ("Exercise Therapies"[Title/Abstract])) OR ("Therapies, Exercise"[Title/Abstract])) OR ("Remedial Exercise"[Title/Abstract])) OR ("Exercise, Remedial"[Title/Abstract])) OR ("Exercises, Remedial"[Title/Abstract])) OR ("Remedial Exercises"[Title/Abstract])) OR ("Muscle Stretching Exercises"[Title/Abstract])) OR ("Exercise, Muscle Stretching"[Title/Abstract])) OR ("Muscle Stretching Exercise"[Title/Abstract])) OR ("Active Stretching"[Title/Abstract])) OR ("Stretching, Active"[Title/Abstract])) OR ("Static-Active Stretching"[Title/Abstract])) OR ("Static Active Stretching"[Title/Abstract])) OR ("Stretching, Static-Active"[Title/Abstract])) OR ("Proprioceptive Neuromuscular Facilitation (PNF) Stretching"[Title/Abstract])) OR ("Proprioceptive Neuromuscular Facilitation"[Title/Abstract])) OR ("Neuromuscular Facilitation, Proprioceptive"[Title/Abstract])) OR ("Proprioceptive Neuromuscular Facilitations"[Title/Abstract])) OR ("PNF Stretching"[Title/Abstract])) OR ("PNF Stretchings"[Title/Abstract])) OR ("Stretching, PNF"[Title/Abstract])) OR ("PNF Stretching Exercise"[Title/Abstract])) OR ("Exercise, PNF Stretching"[Title/Abstract])) OR ("PNF Stretching Exercises"[Title/Abstract])) OR ("Stretching Exercise, PNF"[Title/Abstract])) OR ("Passive Stretching"[Title/Abstract])) OR ("Stretching, Passive"[Title/Abstract])) OR ("Static-Passive Stretching"[Title/Abstract])) OR ("Static Passive Stretching"[Title/Abstract])) OR ("Stretching, Static-Passive"[Title/Abstract])) OR ("Relaxed Stretching"[Title/Abstract])) OR ("Stretching, Relaxed"[Title/Abstract])) OR ("Static Stretching"[Title/Abstract])) OR ("Stretching, Static"[Title/Abstract])) OR ("Isometric Stretching"[Title/Abstract])) OR ("Stretching, Isometric"[Title/Abstract])) OR ("Dynamic Stretching"[Title/Abstract])) OR ("Stretching, Dynamic"[Title/Abstract])) OR ("Ballistic Stretching"[Title/Abstract])) OR ("Stretching, Ballistic"[Title/Abstract])) OR ("Aquatic Therapy"[Title/Abstract])) OR ("Hydrotherapy"[Mesh]) OR ("Therapy, Aquatic"[Title/Abstract])) OR ("Pool Therapy"[Title/Abstract])) OR ("Therapy, Pool"[Title/Abstract])) OR ("Ai Chi Therapy"[Title/Abstract])) OR ("Therapies, Ai Chi"[Title/Abstract])) OR ("Therapy, Ai Chi"[Title/Abstract])) OR ("Water Tai Chi Therapy"[Title/Abstract])) OR ("Aquatic Exercise Therapy"[Title/Abstract])) OR ("Exercise Therapy, Aquatic"[Title/Abstract])) OR ("Therapy, Aquatic Exercise"[Title/Abstract])) OR ("Water Exercise Therapy"[Title/Abstract])) OR ("Exercise Therapy, Water"[Title/Abstract])) OR ("Therapy, Water Exercise"[Title/Abstract])) OR ("Water-based Exercise"[Title/Abstract])) OR ("Aqua"[Title/Abstract])) OR ("Resistance Training"[Title/Abstract])) OR ("Training, Resistance"[Title/Abstract])) OR ("Strength Training"[Title/Abstract])) OR ("Training, Strength"[Title/Abstract])) OR ("Weight-Lifting Strengthening Program"[Title/Abstract])) OR ("Strengthening Programs, Weight-Lifting"[Title/Abstract])) OR ("Strengthening Program, Weight-Lifting"[Title/Abstract])) OR ("Weight Lifting Strengthening Program"[Title/Abstract])) OR ("Weight-Lifting Strengthening Programs"[Title/Abstract])) OR ("Weight-Lifting Exercise Program"[Title/Abstract])) OR ("Exercise Programs, Weight-Lifting"[Title/Abstract])) OR ("Exercise Program, Weight-Lifting"[Title/Abstract])) OR ("Weight Lifting Exercise Program"[Title/Abstract])) OR ("Weight-Lifting Exercise Programs"[Title/Abstract])) OR ("Weight-Bearing Strengthening Program"[Title/Abstract])) OR ("Strengthening Programs, Weight-Bearing"[Title/Abstract])) OR ("Strengthening Program, Weight-Bearing"[Title/Abstract])) OR ("Weight bearing Strengthening Program"[Title/Abstract])) OR ("Weight-Bearing Strengthening Programs"[Title/Abstract])) OR ("Weight-Bearing Exercise Program"[Title/Abstract])) OR ("Exercise Programs, Weight-Bearing"[Title/Abstract])) OR ("Exercise Program, Weight-Bearing"[Title/Abstract])) OR ("Weight bearing Exercise Program"[Title/Abstract])) OR ("Weight-Bearing Exercise Programs"[Title/Abstract])) OR ("Muscle Strength Training"[Title/Abstract])) OR ("Yoga"[Mesh]) OR ("Tai Ji"[Title/Abstract])) OR ("Tai-ji"[Title/Abstract])) OR ("Tai Chi"[Title/Abstract])) OR ("Chi, Tai"[Title/Abstract])) OR ("Tai Ji Quan"[Title/Abstract])) OR ("Ji Quan, Tai"[Title/Abstract])) OR ("Quan, Tai Ji"[Title/Abstract])) OR ("Taiji"[Title/Abstract])) OR ("Taijiquan"[Title/Abstract])) OR ("T'ai Chi"[Title/Abstract])) OR ("Tai Chi Chuan"[Title/Abstract])) OR ("Qigong"[Title/Abstract])) OR ("Qi Gong"[Title/Abstract])) OR ("Ch'i Kung"[Title/Abstract])) OR ("Exercise Movement Techniques"[Title/Abstract])) OR ("Movement Techniques, Exercise"[Title/Abstract])) OR ("Exercise Movement Technics"[Title/Abstract])) OR ("Pilates-Based Exercises"[Title/Abstract])) OR ("Exercises, Pilates-Based"[Title/Abstract])) OR ("Pilates Based Exercises"[Title/Abstract])) OR ("Pilates Training"[Title/Abstract])) OR ("Training, Pilates"[Title/Abstract])) OR ("McKenzie method"[Title/Abstract])) OR ("Heckscher training"[Title/Abstract])) OR ("running"[Title/Abstract])) OR ("jogging"[Title/Abstract])) OR ("exergame"[Title/Abstract])) OR ("Swiss balls"[Title/Abstract])) OR ("Breathing Exercises"[Title/Abstract])) OR ("Inspiratory muscle training"[Title/Abstract])) OR ("Expiratory muscle training"[Title/Abstract])) OR ("Exercise, Breathing"[Title/Abstract])) OR ("Respiratory Muscle Training"[Title/Abstract])) OR ("Muscle Training, Respiratory"[Title/Abstract])) OR ("Training, Respiratory Muscle"[Title/Abstract])) OR ("Exercise"[Mesh]) OR ("High-Intensity Interval Training"[Title/Abstract])) OR ("High Intensity Interval Training"[Title/Abstract])) OR ("High-Intensity Interval Trainings"[Title/Abstract])) OR ("Interval Training, High-Intensity"[Title/Abstract])) OR ("Interval Trainings, High-Intensity"[Title/Abstract])) OR ("Training"[Title/Abstract])) OR ("balance training"[Title/Abstract])) OR ("balance trainings"[Title/Abstract])) OR ("balance exercise"[Title/Abstract])) OR ("balance exercises"[Title/Abstract])) OR ("flexibility training"[Title/Abstract])) OR ("flexibility trainings"[Title/Abstract])) OR ("flexibility exercise"[Title/Abstract])) OR ("flexibility exercises"[Title/Abstract])) OR ("joint mobility training"[Title/Abstract])) OR ("joint mobility trainings"[Title/Abstract])) OR ("joint mobility exercise"[Title/Abstract])) OR ("joint mobility exercises"[Title/Abstract])) OR ("functional movement training"[Title/Abstract])) OR ("functional movement trainings"[Title/Abstract])) OR ("functional movement exercise"[Title/Abstract])) OR ("functional movement exercises"[Title/Abstract])) OR ("stability training"[Title/Abstract])) OR ("stability trainings"[Title/Abstract])) OR ("stability exercise"[Title/Abstract])) OR ("stability exercises"[Title/Abstract])) OR ("medical gymnastics"[Title/Abstract])))) AND (randomized controlled trial[Publication Type] OR randomized[Title/Abstract] OR placebo[Title/Abstract]) Filters: from 1000/1/1 - 2025/12/30

**Web of science**

#1 TS=(Inflammatory Bowel Diseases OR inflammatory bowel disease OR IBD OR Colitis Ulcerative OR ulcerative colitis OR UC OR Crohn Disease OR Inflammatory Bowel Diseases OR inflammatory bowel disease OR IBD OR Colitis Ulcerative OR ulcerative colitis OR UC OR Crohn Disease OR Crohn's disease OR Crohn disease OR regional enteritis OR Crohn's Enteritis OR Regional Enteritis OR Ileocolitis OR Ileitis, Terminal OR Terminal Ileitis OR Ileitis, Regional OR Regional Ileitides OR Regional Ileitis OR Enteritis, Granulomatous OR Granulomatous Enteritis OR Enteritis, Regional OR Colitis, Granulomatous OR Granulomatous ColitisCrohn disease OR regional enteritis OR Crohn's Enteritis OR Regional Enteritis OR Ileocolitis OR Ileitis, Terminal OR Terminal Ileitis OR Ileitis, Regional OR Regional Ileitides OR Regional Ileitis OR Enteritis, Granulomatous OR Granulomatous Enteritis OR Enteritis, Regional OR Colitis, Granulomatous OR Granulomatous Colitis)

2# TS=((Exercise Therapy OR Rehabilitation Exercise OR Exercise, Rehabilitation OR Exercises, Rehabilitation OR Rehabilitation Exercises OR Therapy, Exercise OR Exercise Therapies OR Therapies, Exercise OR Remedial Exercise OR Exercise, Remedial OR Exercises, Remedial OR Remedial Exercises) OR (Muscle Stretching Exercises OR Exercise, Muscle Stretching OR Muscle Stretching Exercise OR Active Stretching OR Stretching, Active OR Static-Active Stretching OR Static Active Stretching OR Stretching, Static-Active OR Proprioceptive Neuromuscular Facilitation (PNF) Stretching OR Proprioceptive Neuromuscular Facilitation OR Neuromuscular Facilitation, Proprioceptive OR Proprioceptive Neuromuscular Facilitations OR PNF Stretching OR PNF Stretchings OR Stretching, PNF OR PNF Stretching Exercise OR Exercise, PNF Stretching OR PNF Stretching Exercises OR Stretching Exercise, PNF OR Passive Stretching OR Stretching, Passive OR Static-Passive Stretching OR Static Passive Stretching OR Stretching, Static-Passive OR Relaxed Stretching OR Stretching, Relaxed OR Static Stretching OR Stretching, Static OR Isometric Stretching OR Stretching, Isometric OR Dynamic Stretching OR Stretching, Dynamic OR Ballistic Stretching OR Stretching, Ballistic) OR (Aquatic Therapy OR Hydrotherapy OR Therapy, Aquatic OR Pool Therapy OR Therapy, Pool OR Ai Chi Therapy OR Therapies, Ai Chi OR Therapy, Ai Chi OR Water Tai Chi Therapy OR Aquatic Exercise Therapy OR Exercise Therapy, Aquatic OR Therapy, Aquatic Exercise OR Water Exercise Therapy OR Exercise Therapy, Water OR Therapy, Water Exercise OR Water-based Exercise OR Aqua) OR (Resistance Training OR Training, Resistance OR Strength Training OR Training, Strength OR Weight-Lifting Strengthening Program OR Strengthening Programs, Weight-Lifting OR Strengthening Program, Weight-Lifting OR Weight Lifting Strengthening Program OR Weight-Lifting Strengthening Programs OR Weight-Lifting Exercise Program OR Exercise Programs, Weight-Lifting OR Exercise Program, Weight-Lifting OR Weight Lifting Exercise Program OR Weight-Lifting Exercise Programs OR Weight-Bearing Strengthening Program OR Strengthening Programs, Weight-Bearing OR Strengthening Program, Weight-Bearing OR Weight bearing Strengthening Program OR Weight-Bearing Strengthening Programs OR Weight-Bearing Exercise Program OR Exercise Programs, Weight-Bearing OR Exercise Program, Weight-Bearing OR Weight bearing Exercise Program OR Weight-Bearing Exercise Programs OR Muscle Strength Training) OR ("Yoga" OR "Tai Ji" OR Tai-ji OR Tai Chi OR Chi, Tai OR Tai Ji Quan OR Ji Quan, Tai OR Quan, Tai Ji OR Taiji OR Taijiquan OR "T'ai Chi" OR Tai Chi Chuan OR "Qigong" OR Qi Gong OR Ch'i Kung OR "Exercise Movement Techniques" OR Movement Techniques, Exercise OR Exercise Movement Technics OR Pilates-Based Exercises OR Exercises, Pilates-Based OR Pilates Based Exercises OR Pilates Training OR Training, Pilates OR McKenzie method OR Heckscher training OR running OR jogging OR exergame OR Swiss balls) OR ("Breathing Exercises" OR Inspiratory muscle training OR Expiratory muscle training OR Exercise, Breathing OR Respiratory Muscle Training OR Muscle Training, Respiratory OR Training, Respiratory Muscle) OR ("Exercise" OR "High-Intensity Interval Training" OR High Intensity Interval Training OR High-Intensity Interval Trainings OR Interval Training, High-Intensity OR Interval Trainings, High-Intensity OR Training) OR (balance training OR balance trainings OR balance exercise OR balance exercises OR flexibility training OR flexibility trainings OR flexibility exercise OR flexibility exercises OR joint mobility training OR joint mobility trainings OR joint mobility exercise OR joint mobility exercises OR functional movement training OR functional movement trainings OR functional movement exercise OR functional movement exercises OR stability training OR stability trainings OR stability exercise OR stability exercises OR medical gymnastics))

3# TS=(randomized controlled trial OR controlled clinical trial OR randomized OR placebo OR clinical trials as topic OR RCT)

Result：#1 AND #2 AND #3

**Cochrane library**

#1 (Inflammatory Bowel Diseases):ab,ti,kw OR (inflammatory bowel disease):ab,ti,kw OR (IBD):ab,ti,kw OR (Colitis Ulcerative):ab,ti,kw OR (ulcerative colitis):ab,ti,kw OR (UC):ab,ti,kw OR (Crohn Disease):ab,ti,kw OR (Crohn's disease):ab,ti,kw OR (Crohn disease):ab,ti,kw OR (regional enteritis):ab,ti,kw OR (Crohn's Enteritis):ab,ti,kw OR (Regional Enteritis):ab,ti,kw OR (Ileocolitis):ab,ti,kw OR (Ileitis, Terminal):ab,ti,kw OR (Terminal Ileitis):ab,ti,kw OR (Ileitis, Regional):ab,ti,kw OR (Regional Ileitides):ab,ti,kw OR (Regional Ileitis):ab,ti,kw OR (Enteritis, Granulomatous):ab,ti,kw OR (Granulomatous Enteritis):ab,ti,kw OR (Enteritis, Regional):ab,ti,kw OR (Colitis, Granulomatous):ab,ti,kw OR (Granulomatous Colitis):ab,ti,kw

#3 #1AND #2

( (Exercise Therapy OR Rehabilitation Exercise OR Remedial Exercise OR "Exercise Therapies" OR "Rehabilitation Exercises") OR (Muscle Stretching Exercises OR "Active Stretching" OR "Static Active Stretching" OR "Proprioceptive Neuromuscular Facilitation Stretching" OR "PNF Stretching" OR "Passive Stretching" OR "Static Passive Stretching" OR "Relaxed Stretching" OR "Static Stretching" OR "Isometric Stretching" OR "Dynamic Stretching" OR "Ballistic Stretching") OR (Aquatic Therapy OR Hydrotherapy OR "Pool Therapy" OR "Ai Chi Therapy" OR "Water Tai Chi Therapy" OR "Aquatic Exercise Therapy" OR "Water Exercise Therapy" OR "Water based Exercise" OR Aqua) OR (Resistance Training OR Strength Training OR "Weight Lifting Strengthening Program" OR "Weight Bearing Strengthening Program" OR "Muscle Strength Training") OR ("Yoga" OR "Tai Ji" OR "Tai Chi" OR "Tai Ji Quan" OR "Taijiquan" OR "T'ai Chi" OR "Qigong" OR "Exercise Movement Techniques" OR "Pilates Based Exercises" OR "Pilates Training" OR "McKenzie method" OR "Heckscher training" OR running OR jogging OR exergame OR "Swiss balls") OR ("Breathing Exercises" OR "Inspiratory muscle training" OR "Expiratory muscle training" OR "Respiratory Muscle Training") OR (Exercise OR "High Intensity Interval Training" OR Training) OR (balance training OR balance exercise OR flexibility training OR flexibility exercise OR "joint mobility training" OR "joint mobility exercise" OR "functional movement training" OR "functional movement exercise" OR stability training OR stability exercise OR "medical gymnastics") ) AND (randomized controlled trial OR controlled clinical trial OR randomized OR placebo OR "clinical trials as topic" OR RCT)

**Embase**

#1 :'inflammatory bowel diseases':ab,ti OR 'inflammatory bowel disease':ab,ti OR 'ibd':ab,ti OR 'colitis ulcerative':ab,ti OR 'ulcerative colitis':ab,ti OR 'uc':ab,ti OR 'crohn disease':ab,ti OR 'crohn enteritis':ab,ti OR 'regional enteritis':ab,ti OR 'ileocolitis':ab,ti OR 'ileitis, terminal':ab,ti OR 'terminal ileitis':ab,ti OR 'ileitis, regional':ab,ti OR 'regional ileitides':ab,ti OR 'regional ileitis':ab,ti OR 'enteritis, granulomatous':ab,ti OR 'granulomatous enteritis':ab,ti OR 'enteritis, regional':ab,ti OR 'colitis, granulomatous':ab,ti OR 'granulomatous colitis':ab,ti

#2:'rehabilitation exercise*':ab,ti,kw OR 'rehabilitation exercises':ab,ti,kw OR 'exercises, rehabilitation':ab,ti,kw OR 'exercises rehabilitation':ab,ti,kw OR 'rehabilitation therapies':ab,ti,kw OR 'therapies, exercise':ab,ti,kw OR 'exercise therapy*':ab,ti,kw OR 'exercise therapies':ab,ti,kw OR 'remedial exercise*':ab,ti,kw OR 'remedial exercises':ab,ti,kw OR 'exercise, remedial':ab,ti,kw OR 'exercises, remedial':ab,ti,kw OR 'muscle stretching exercise*':ab,ti,kw OR 'muscle stretching exercises':ab,ti,kw OR 'exercise, muscle stretching':ab,ti,kw OR 'active stretching':ab,ti,kw OR 'stretching, active':ab,ti,kw OR 'static-active stretching':ab,ti,kw OR 'static active stretching':ab,ti,kw OR 'stretching, static-active':ab,ti,kw OR 'proprioceptive neuromuscular facilitation (pnf) stretching':ab,ti,kw OR 'proprioceptive neuromuscular facilitation':ab,ti,kw OR 'neuromuscular facilitation, proprioceptive':ab,ti,kw OR 'proprioceptive neuromuscular facilitations':ab,ti,kw OR 'pnf stretching':ab,ti,kw OR 'pnf stretchings':ab,ti,kw OR 'stretching, pnf':ab,ti,kw OR 'pnf stretching exercise':ab,ti,kw OR 'exercise, pnf stretching':ab,ti,kw OR 'pnf stretching exercises':ab,ti,kw OR 'stretching exercise, pnf':ab,ti,kw OR 'passive stretching':ab,ti,kw OR 'stretching, passive':ab,ti,kw OR 'static-passive stretching':ab,ti,kw OR 'static passive stretching':ab,ti,kw OR 'stretching, static-passive':ab,ti,kw OR 'relaxed stretching':ab,ti,kw OR 'stretching, relaxed':ab,ti,kw OR 'static stretching':ab,ti,kw OR 'stretching, static':ab,ti,kw OR 'isometric stretching':ab,ti,kw OR 'stretching, isometric':ab,ti,kw OR 'dynamic stretching':ab,ti,kw OR 'stretching, dynamic':ab,ti,kw OR 'ballistic stretching':ab,ti,kw OR 'stretching, ballistic':ab,ti,kw OR 'aquatic therapy':ab,ti,kw OR 'hydrotherapy':ab,ti,kw OR 'therapy, aquatic':ab,ti,kw OR 'pool therapy':ab,ti,kw OR 'therapy, pool':ab,ti,kw OR 'ai chi therapy':ab,ti,kw OR 'therapies, ai chi':ab,ti,kw OR 'therapy, ai chi':ab,ti,kw OR 'water tai chi therapy':ab,ti,kw OR 'aquatic exercise therapy':ab,ti,kw OR 'exercise therapy, aquatic':ab,ti,kw OR 'therapy, aquatic exercise':ab,ti,kw OR 'water exercise therapy':ab,ti,kw OR 'exercise therapy, water':ab,ti,kw OR 'therapy, water exercise':ab,ti,kw OR 'water-based exercise':ab,ti,kw OR 'aqua':ab,ti,kw OR 'resistance training':ab,ti,kw OR 'training, resistance':ab,ti,kw OR 'strength training':ab,ti,kw OR 'training, strength':ab,ti,kw OR 'weight-lifting strengthening program*':ab,ti,kw OR 'strengthening programs, weight-lifting':ab,ti,kw OR 'strengthening program, weight-lifting':ab,ti,kw OR 'weight lifting strengthening program':ab,ti,kw OR 'weight-lifting strengthening programs':ab,ti,kw OR 'weight-lifting exercise program*':ab,ti,kw OR 'exercise programs, weight-lifting':ab,ti,kw OR 'exercise program, weight-lifting':ab,ti,kw OR 'weight lifting exercise program':ab,ti,kw OR 'weight-lifting exercise programs':ab,ti,kw OR 'weight-bearing strengthening program*':ab,ti,kw OR 'strengthening programs, weight-bearing':ab,ti,kw OR 'strengthening program, weight-bearing':ab,ti,kw OR 'weight bearing strengthening program':ab,ti,kw OR 'weight-bearing strengthening programs':ab,ti,kw OR 'weight-bearing exercise program*':ab,ti,kw OR 'exercise programs, weight-bearing':ab,ti,kw OR 'exercise program, weight-bearing':ab,ti,kw OR 'weight bearing exercise program':ab,ti,kw OR 'weight-bearing exercise programs':ab,ti,kw OR 'muscle strength training':ab,ti,kw OR 'yoga':ab,ti,kw OR 'tai ji':ab,ti,kw OR 'tai-ji':ab,ti,kw OR 'chi, tai':ab,ti,kw OR 'tai ji quan':ab,ti,kw OR 'ji quan, tai':ab,ti,kw OR 'quan, tai ji':ab,ti,kw OR 'taiji':ab,ti,kw OR 'taijiquan':ab,ti,kw OR 'tai chi':ab,ti,kw OR 'tai chi chuan':ab,ti,kw OR 'qigong':ab,ti,kw OR 'qi gong':ab,ti,kw OR 'chi kung':ab,ti,kw OR 'exercise movement techniques':ab,ti,kw OR 'movement techniques, exercise':ab,ti,kw OR 'exercise movement technics':ab,ti,kw OR 'pilates-based exercises':ab,ti,kw OR 'exercises, pilates-based':ab,ti,kw OR 'pilates based exercises':ab,ti,kw OR 'pilates training':ab,ti,kw OR 'training, pilates':ab,ti,kw OR 'mckenzie method':ab,ti,kw OR 'heckscher training':ab,ti,kw OR 'running':ab,ti,kw OR 'jogging':ab,ti,kw OR 'exergame':ab,ti,kw OR 'swiss balls':ab,ti,kw OR 'breathing exercises':ab,ti,kw OR 'inspiratory muscle training':ab,ti,kw OR 'expiratory muscle training':ab,ti,kw OR 'exercise, breathing':ab,ti,kw OR 'respiratory muscle training':ab,ti,kw OR 'muscle training, respiratory':ab,ti,kw OR 'training, respiratory muscle':ab,ti,kw OR 'exercise':ab,ti,kw OR 'high-intensity interval training':ab,ti,kw OR 'high intensity interval training':ab,ti,kw OR 'high-intensity interval trainings':ab,ti,kw OR 'interval training, high-intensity':ab,ti,kw OR 'interval trainings, high-intensity':ab,ti,kw OR 'training':ab,ti,kw OR 'balance training':ab,ti,kw OR 'balance trainings':ab,ti,kw OR 'balance exercise':ab,ti,kw OR 'balance exercises':ab,ti,kw OR 'flexibility training':ab,ti,kw OR 'flexibility trainings':ab,ti,kw OR 'flexibility exercise':ab,ti,kw OR 'flexibility exercises':ab,ti,kw OR 'joint mobility training':ab,ti,kw OR 'joint mobility trainings':ab,ti,kw OR 'joint mobility exercise':ab,ti,kw OR 'joint mobility exercises':ab,ti,kw OR 'functional movement training':ab,ti,kw OR 'functional movement trainings':ab,ti,kw OR 'functional movement exercise':ab,ti,kw OR 'functional movement exercises':ab,ti,kw OR 'stability training':ab,ti,kw OR 'stability trainings':ab,ti,kw OR 'stability exercise':ab,ti,kw OR 'stability exercises':ab,ti,kw OR 'medical gymnastics':ab,ti,kw

#3:randomized*:ab,ti,kw OR controlled) AND clinical AND trial*:ab,ti,kw OR random*:ab,ti,kw OR placebo*:ab,ti,kw OR rct:ab,ti,kw

**CNKI**

（主题：炎症性肠病 + 溃疡性结肠炎 + 克罗恩病）AND（主题：运动 + 运动疗法 + 瑜伽 + 有氧运动 + 太极 + 高强度间歇运动 + 抗阻训练 + 联合运动）

**万方学术期刊全文数据库**

(主题:(炎症性肠病 OR 溃疡性结肠炎 OR 克罗恩病) and 主题:(运动 OR 运动疗法 OR 瑜伽 OR 有氧运动 OR 太极 OR 高强度间歇运动 OR 抗阻训练 OR 联合运动))

**Supplementary Figures：**

Figure S1 Risk of bias assessment included in the study.

Figure S2:Comparative Analysis of HIT versus Conventional Therapy in the Quality of Life Subgroup

Figure S3:Comparative Analysis of CAE versus Conventional Therapy in the Quality of Life Subgroup

Figure S4：Comparative Analysis of CAE versus HIT in the Quality of Life Subgroup

Figure S5:Heterogeneity in the CAE subgroup analysis of quality of life after exclusion of Chinese studies

Figure S6:Sensitivity analysis of CAE for improving quality of life

Figure S7：Subgroup analysis of CAE for quality of life and meta-regression analysis by different country

Figure S8：Subgroup analysis of CAE for quality of life and meta-regression analysis by different frequencies

Figure S9：Subgroup analysis of CAE for quality of life and meta-regression analysis by different duration time

Figure S10：Subgroup analysis of the effect of sustained aerobic exercise on fatigue level

Figure S11：Subgroup analysis of the effects of different exercise modalities on fatigue level

Figure S12：Subgroup analysis of the effects of different exercise modalities on C-reactive protein (CRP)

Figure S13:Subgroup analysis of the effects of different exercise modalities on ESR

Figure S14:	Subgroup analysis of the effect of CAE across different countries on quality of life

Figure S15：Subgroup analysis of the effect of different city of CAE on quality of life

Figure S16：Subgroup analysis of the effect of different intervention frequencies of CAE on quality of life

Figure S17：Subgroup analysis of the effect of different intervention timings of CAE on quality of life

Figure S18：Subgroup analysis of the effect of different intervention timings of CAE on fatigue severity

Figure S19：	Subgroup analysis of the effect of CAE across different countries on fatigue severity

Figure S20：Sensitivity analysis of CAE for improving fatigue severity

Figure S21：Subgroup analysis of CAE for fatigue severity and meta-regression analysis by different country

Figure S22：Subgroup analysis of CAE for fatigue severity and meta-regression analysis by different time

**Supplementary Table S1** Basic information of all the papers

（2）CE:combined aerobic and resistance exercise;（3）CAE:Continuous aerobic exercise;（4）yoga:;（5）TC:Tai Chi;（6）RE:Resistance exercise;

（7）HIIT:High-intensity interval training

Note: CT:Conventional Therapy;

①Quality of Life;②CAI;③CRP;④:fatigue;⑤:ESR;⑥:IL-6;⑦:TNF-α

| Paper | Diagnostic  Criteria | Sample size (Control /Treatment  /Other) | Gender  (M/F) | Control group | Treatment group | Other group | Intervention  Control group | Intervention  Treatment group | Intervention  Other group | age(years)  Control group | age(years)  Treatment group | age(years)  Other group | Duration of treatment(month） | Outcomes | randomization method |
| --- | --- | --- | --- | --- | --- | --- | --- | --- | --- | --- | --- | --- | --- | --- | --- |
| Katherine  2020 | Aged≥16,Stool calprotein of <250μg/g,stable medication(>4w),quiescent or mildly-active disease,  CDAI<150 or 150-219 | 24/23 | 15/32 | 21/3 | 22/1 | — | CT | RE | — | 52.3±13.6 | 46.1±11.9 | — | 3  （3 per week，60min） | ①④ | Automated Online Randomization Program |
| H.Cramer  2017 | Aged:18–70, endoscopic and histological diagnosed UC >12 months,In clinical remission phase>4 weeks but≤12month; RI≤4;IBDQ<170 | 38/39 | 19/58 | 38/0 | 39/0 | — | CT | Yoga | — | 46.1±10.4 | 45.0±13.3 | — | 3 | ①② | Blocked Randomization |
| Hu Yalin  2021 | Aged≥18,endoscopic  and histological diagnosed UC/CD,in mild to moderate or remission  phase | 35/35 | 48/22 | 30/5 | 32/3 | — | CT | yoga | — | — | 33. 16 ± 10. 40 | 37. 53 ± 11. 34 | 6 | ③④⑤ | Random |
| Peter Klare  2014 | Aged≥18,diagnosed CD or UC at least one year,phase: in remission or with mild active  (Crohn‘s Disease Activity Index (CDAI) <220 points or Rachmilewitz Index (RI) <11 points | 15/15 | 8/22 | 15/0 | 15/0 | — | CT | CAE | — | — | 42.5±13.9 | 39.7±14.7 | 2.5  （3 per week） | ① | Random |
| R. Izhar  2024 | unclear | 11/15 | — | 11/0 | 15/0 | — | CT | yoga | — | — | — | — | 1 | ② | Random |
| Victor  Ng,MSc  2007 | unclear | 16/16 | 14/18 | 16/0 | 16/0 | — | CT | CAE | — | 37.0±12.7 | 40.6±11.7 | — | 3  （3/week30min） | ① | Random |
| Tao MQ2022 | Aged:18–60;diagnosed CD or UC,in remission(The mayo score of UC ≤ 2;CDAI<150);ability to walk but no regular exercise habit on a daily basis | 51/49 | 41/59 | 51/0 | 49/0 | — | CT | CAE | — | 42.7±12.9 | 39.8±13.6 | — | 3 | ①④ | Random Number |
| Tao MQ2023 | Aged≥18;diagnosed CD or UC;in remission phase | 33/34 | 35/32 | 33/0 | 34/0 | — | CT | CAE | — | 47.91±9.42 | 45.43±9.77 | — | 3 | ④ | Random Number |
| JiangH  2023 | endoscopic and histological diagnosed CD or UC≥1 year;in remission phase;no regular exercise habit or exercise <2 hours per week;Vitals stable | 60/60 | 35/25 | 57/3 | 58/2 | — | CT | CAE | — | 36.45±12.69 | 36.57±12.73 | — | 3 | ④⑥⑦ |  |
| ShiML  2014 | Aged:16-65;endoscopic and histological diagnosed;in remission phase | 16/20 | 13/23 | 16/0 | 20/0 | — | CT | CAE | — | 42.7±3.8 | 45.5±4.6 | — | 4 | ⑥⑦ |  |
| DingWQ  2018 | endoscopic and histological diagnosed IBD(CDA<220 or RI<11);in remission phase after regular treatment | 28/28 | — | 26/2 | 24/4 | — | CT | CAE | — | 41.5±13.8 | 38.6±14.7 | — | 2.5 | ① | Random |
| HuangZF  2025 | endoscopic and histological diagnosed CD;Best-CDAI<150 | 8/14 | 8/14 | 8/0 | 14/0 | — | CT | TaiChi | — | 37.48±7.61 | 37.53±7.24 | — | 3 | ②③⑤ | Random Number |
| DingWQ  2024 | Aged:18-60;endoscopic and histological diagnosed IBD>1 year;in symptomatic remission or mildly active phase | 35/35 | 25/38 | 32/3 | 31/4 | — | CT | CE | — | 37.56±11.68 | 34.19±10.91 | — | 3 | ①④ | Random |
| HuQQ  2020 | Aged≥18;endoscopic and histological diagnosed IBD | 45/46 | — | 45/0 | 46/0 | — | CT | CE | — | — | — | — | 1 | ① | Random |
| Pan YH  2025 | endoscopic and histological diagnosed UC | 61/61 | 62/60 | 61/0 | 61/0 | — | CT | HIT | — | 32.23±9.35 | 34.32±9.56 | — | 3 | ⑥⑦ | Random |
| LiZL  2019 | Aged:18-65;endoscopic and histological diagnosed UC;no regular exercise>3 month;Steroid-dependent or refractory | 52/52 | 62/42 | 52/0 | 52/0 | — | CT | HIT | — | 49.52±5.71 | 49.38±5.74 | — | 1 | ⑥⑦ | Random |
| XieF  2020 | endoscopic and histological diagnosed UC | 32/32 | 35/29 | 32/0 | 32/0 | — | CT | HIT | — | 58.36±2.14 | 58.42±2.33 |  | 2 | ⑥⑦ | Random |
| WolfgangAS 2020 | Aged:18–65;diagnosed with CD≥6 months;in symptomatic remission or mildly active phase  And stable medication≥4 weeks | CAE=17  RE=15  CT=13 | 26/19 | 12/1 | 12/3 | 11/6 | CT | RE | CAE | 43.7±12.0 | 45.0±11.3 | 45.3±12.4 | 3 | ② | Random |
| Garry A  2019 | Aged:18–65;diagnosed CD;FCP<250ug/g;stable medication≥4 weeks;in symptomatic remission or mildly active phase | HIT=13  CAE=12  CT=11 | 17/30 | 10/1 | 11/2 | 11/1 | CT | HIT | CAE | 35.0±10.0 | 37.0±11.1 | 38.5±13.0 | 3 | ①②④ | Random |
| ZhangY  2016 | diagnosed UC;in symptomatic remission phase | HIT=20  CAE=20  CT=17 | 30/27 | 16/1 | 16/4 | 14/6 | CT | HIT | CAE | 47.1±5.6 | 44.7±4.0 | 46.2±5.1 | 3 | ① | Random |
| WuZW  2017 | unclear | 28/28 | 33/23 | 28/0 | 28/0 | — | CAE | HIT | — | 41.2±5.6 | 42.5±6.2 | — | 3 | ① | Random |
| LiuYH  2018 | unclear | 55/65 | 65/55 | 55/0 | 65/0 | — | CAE | HIT | — | 43.46±12.39 | 43.18±12.65 | — | 3 | ① | Random |
| ShiZJ  2020 | unclear | 40/40 | 37/43 | 40/0 | 40/0 | — | CAE | HIT | — | 48.92±5.66 | 48.95±5.21 | — | 3 | ① | Random |
| Patricia L. Gerbarg  2015 | Aged:18-85;diagnosed IBD | 14/15 | 12/17 | 14/0 | 15/0 | — | CT | TC | — | 58.57±16.22 | 49.27±14.21 | — | 7 | ① | Random Number |

**Supplementary Table S2** Results of network Meta-Analysis(IBDQ)

| **Interventions** | **CT** | **CE** | **RE** | **Yoga** | **TC** | **HIT** | **CAE** |
| --- | --- | --- | --- | --- | --- | --- | --- |
| **CAE** | 1.15 (0.29,2.01) | 0.74 (-1.03,2.51) | 0.65 (-1.73,3.04) | 0.63 (-1.72,2.99) | 0.30 (-2.13,2.73) | 0.03 (-1.20,1.26) | 0 |
| **HIT** | 1.12 (-0.11,2.36) | 0.71 (-1.27,2.69) | 0.63 (-1.92,3.17) | 0.61 (-1.91,3.12) | 0.27 (-2.31,2.86) | 0 |  |
| **TC** | 0.85 (-1.42,3.12) | 0.44 (-2.32,3.19) | 0.35 (-2.83,3.53) | 0.33 (-2.82,3.49) | 0 |  |  |
| **Yoga** | 0.52 (-1.67,2.71) | 0.10 (-2.58,2.78) | 0.02 (-3.10,3.14) | 0 |  |  |  |
| **RE** | 0.50 (-1.73,2.72) | 0.08 (-2.63,2.80) | 0 |  |  |  |  |
| **CE** | 0.41 (-1.13,1.96) | 0 |  |  |  |  |  |
| **CT** | 0 |  |  |  |  |  |  |

**Supplementary Table S3** Results of network Meta-Analysis(CDAI)

| **Interventions** | **CT** | **RE** | **CAE** | **Yoga** | **HIT** | **TC** |
| --- | --- | --- | --- | --- | --- | --- |
| **TC** | -1.00 (-2.04,0.03) | -0.63 (-1.88,0.61) | -0.60 (-1.74,0.54) | -0.39  (-1.56,0.78) | -0.13  (-1.41,1.14) | 0 |
| **HIT** | -0.87 (-1.61,-0.13) | -0.50 (-1.46,0.46) | -0.46 (-1.20,0.27) | -0.26 (-1.17,0.66) | 0 |  |
| **Yoga** | -0.61 (-1.15,-0.07) | -0.03 (-0.74,0.67) | -0.21 (-0.92,0.50) | 0 |  |  |
| **CAE** | -0.40 (-0.88,0.07) | -0.03 (-0.74,0.67) | 0 |  |  |  |
| **RE** | -0.37 (-1.06,0.33) | 0 |  |  |  |  |
| **CT** | 0 |  |  |  |  |  |

| **Interventions** | **CT** | **HIT** | **RE** | **CAE** | **Yoga** |
| --- | --- | --- | --- | --- | --- |
| **Yoga** | -3.44 (-6.63,-0.25) | -3.01 (-6.98,0.95) | -2.96 (-7.44,1.52) | -2.33 (-5.79,1.13) | 0 |
| **CAE** | -1.11 (-2.45,0.24) | -0.68 (-3.04,1.68) | -0.63 (-4.05,2.79) | 0 |  |
| **RE** | -0.48 (-3.62,2.67) | -0.05 (-3.98,3.88) | 0 |  |  |
| **HIT** | -0.43 (-2.79,1.94) | 0 |  |  |  |
| **CT** | 0 |  |  |  |  |

**Supplementary Table S4** Results of network Meta-Analysis(IBD-F)

**Supplementary Table S5** Results of network Meta-Analysis(IL-6)

| **Interventions** | **CT** | **CAE** | **HIT** |
| --- | --- | --- | --- |
| **HIT** | -12.81  (-19.90,-5.73) | -6.64  (-17.85,4.58) | 0 |
| **CAE** | -6.18  (-14.87,2.52) | 0 |  |
| **CT** | 0 |  |  |

**Supplementary Table S6** Results of network Meta-Analysis(TNF-α)

Supplementary Table S7 - GRADE assessment of certainty of evidence for the principal outcomes

| **Interventions** | **CT** | **CAE** | **HIT** |
| --- | --- | --- | --- |
| **HIT** | -15.40  (-26.86,-3.94) | -1.81  (-24.81,21.20) | 0 |
| **CAE** | -13.59  (-33.54,6.36) | 0 |  |
| **CT** | 0 |  |  |

**Supplementary Table S7. CINeMA confidence assessment for IBDQ**

*Random-effects network meta-analysis; SMD; equivalence margin: -0.20 to 0.20. Judgments reproduce the final all-low-risk scenario.*

| **Comparison** | **N of studies** | **Within-study bias** | **Reporting bias** | **Indirectness** | **Imprecision** | **Heterogeneity** | **Incoherence** | **Confidence rating** |
| --- | --- | --- | --- | --- | --- | --- | --- | --- |
| CT vs CE | 2 | No concerns | Low risk | No concerns | No concerns | No concerns | Major concerns | Low |
| CT vs CAE | 5 | No concerns | Low risk | No concerns | No concerns | Some concerns | No concerns | Moderate |
| CT vs Yoga | 1 | No concerns | Low risk | No concerns | Major concerns | Some concerns | Some concerns | Very low |
| CT vs TC | 1 | No concerns | Low risk | No concerns | Major concerns | No concerns | Major concerns | Very low |
| CT vs RE | 1 | No concerns | Low risk | No concerns | Some concerns | No concerns | No concerns | Moderate |
| CT vs HIT | 2 | No concerns | Low risk | No concerns | Some concerns | No concerns | No concerns | Moderate |
| CAE vs HIT | 5 | No concerns | Low risk | No concerns | Major concerns | No concerns | No concerns | Low |
| CE vs CAE | 0 | No concerns | Low risk | No concerns | Major concerns | No concerns | Major concerns | Very low |
| CE vs Yoga | 0 | No concerns | Low risk | No concerns | Major concerns | No concerns | Major concerns | Very low |
| CE vs TC | 0 | No concerns | Low risk | No concerns | Major concerns | No concerns | Major concerns | Very low |
| CE vs RE | 0 | No concerns | Low risk | No concerns | Major concerns | No concerns | Major concerns | Very low |
| CE vs HIT | 0 | No concerns | Low risk | No concerns | Major concerns | No concerns | Major concerns | Very low |
| CAE vs Yoga | 0 | No concerns | Low risk | No concerns | Major concerns | No concerns | Major concerns | Very low |
| CAE vs TC | 0 | No concerns | Low risk | No concerns | Major concerns | No concerns | Major concerns | Very low |
| CAE vs RE | 0 | No concerns | Low risk | No concerns | Major concerns | No concerns | Major concerns | Very low |
| Yoga vs TC | 0 | No concerns | Low risk | No concerns | Major concerns | No concerns | Major concerns | Very low |
| Yoga vs RE | 0 | No concerns | Low risk | No concerns | Major concerns | No concerns | Major concerns | Very low |
| Yoga vs HIT | 0 | No concerns | Low risk | No concerns | Major concerns | No concerns | Major concerns | Very low |
| TC vs RE | 0 | No concerns | Low risk | No concerns | Major concerns | No concerns | Major concerns | Very low |
| TC vs HIT | 0 | No concerns | Low risk | No concerns | Major concerns | No concerns | Major concerns | Very low |
| RE vs HIT | 0 | No concerns | Low risk | No concerns | Major concerns | No concerns | Major concerns | Very low |

Note: CT, conventional therapy; CE, combined aerobic and resistance exercise; CAE, continuous aerobic exercise; TC, Tai Chi; RE, resistance exercise; HIT, high-intensity interval training.

**Supplementary Table S8. CINeMA confidence assessment for CDAI**

*Random-effects network meta-analysis; SMD; equivalence margin: -0.20 to 0.20. Judgments reproduce the final all-low-risk scenario.*

| **Comparison** | **N of studies** | **Within-study bias** | **Reporting bias** | **Indirectness** | **Imprecision** | **Heterogeneity** | **Incoherence** | **Confidence rating** |
| --- | --- | --- | --- | --- | --- | --- | --- | --- |
| CT vs CAE | 3 | No concerns | Low risk | No concerns | Some concerns | No concerns | No concerns | Moderate |
| CT vs Yoga | 2 | No concerns | Low risk | No concerns | Major concerns | No concerns | No concerns | Low |
| CT vs TC | 1 | No concerns | Low risk | No concerns | Some concerns | Some concerns | Some concerns | Low |
| CT vs RE | 1 | No concerns | Low risk | No concerns | Major concerns | No concerns | No concerns | Low |
| CT vs HIT | 1 | No concerns | Low risk | No concerns | Some concerns | No concerns | No concerns | Moderate |
| CAE vs RE | 1 | No concerns | Low risk | No concerns | Major concerns | No concerns | No concerns | Low |
| CAE vs HIT | 1 | No concerns | Low risk | No concerns | Some concerns | Some concerns | No concerns | Low |
| CAE vs Yoga | 0 | No concerns | Low risk | No concerns | Major concerns | No concerns | Major concerns | Very low |
| CAE vs TC | 0 | No concerns | Low risk | No concerns | Some concerns | Some concerns | Major concerns | Very low |
| Yoga vs TC | 0 | No concerns | Low risk | No concerns | Major concerns | No concerns | Major concerns | Very low |
| Yoga vs RE | 0 | No concerns | Low risk | No concerns | Major concerns | No concerns | Major concerns | Very low |
| Yoga vs HIT | 0 | No concerns | Low risk | No concerns | Major concerns | No concerns | Major concerns | Very low |
| TC vs RE | 0 | No concerns | Low risk | No concerns | Major concerns | No concerns | Major concerns | Very low |
| TC vs HIT | 0 | No concerns | Low risk | No concerns | Major concerns | No concerns | Major concerns | Very low |
| RE vs HIT | 0 | No concerns | Low risk | No concerns | Major concerns | No concerns | Major concerns | Very low |

Note: CT, conventional therapy; CE, combined aerobic and resistance exercise; CAE, continuous aerobic exercise; TC, Tai Chi; RE, resistance exercise; HIT, high-intensity interval training.

**Supplementary Table S9. CINeMA confidence assessment for IBD-F**

*Random-effects network meta-analysis; SMD; equivalence margin: -0.20 to 0.20. Judgments reproduce the final all-low-risk scenario.*

| **Comparison** | **N of studies** | **Within-study bias** | **Reporting bias** | **Indirectness** | **Imprecision** | **Heterogeneity** | **Incoherence** | **Confidence rating** |
| --- | --- | --- | --- | --- | --- | --- | --- | --- |
| CT vs CAE | 5 | No concerns | Low risk | No concerns | No concerns | Some concerns | Some concerns | Moderate |
| CT vs Yoga | 1 | No concerns | Low risk | No concerns | No concerns | Major concerns | Major concerns | Low |
| CT vs RE | 1 | No concerns | Low risk | No concerns | Major concerns | No concerns | Major concerns | Very low |
| CT vs HIT | 1 | No concerns | Low risk | No concerns | Major concerns | No concerns | No concerns | Low |
| CAE vs HIT | 1 | No concerns | Low risk | No concerns | Major concerns | No concerns | No concerns | Low |
| CAE vs Yoga | 0 | No concerns | Low risk | No concerns | Major concerns | No concerns | Major concerns | Very low |
| CAE vs RE | 0 | No concerns | Low risk | No concerns | Major concerns | No concerns | Major concerns | Very low |
| Yoga vs RE | 0 | No concerns | Low risk | No concerns | Major concerns | No concerns | Major concerns | Very low |
| Yoga vs HIT | 0 | No concerns | Low risk | No concerns | Major concerns | No concerns | Major concerns | Very low |
| RE vs HIT | 0 | No concerns | Low risk | No concerns | Major concerns | No concerns | Major concerns | Very low |

Note: CT, conventional therapy; CE, combined aerobic and resistance exercise; CAE, continuous aerobic exercise; TC, Tai Chi; RE, resistance exercise; HIT, high-intensity interval training.

**Supplementary Table S10. CINeMA confidence assessment for Interleukin-6 (IL-6)**

*Random-effects network meta-analysis; MD; equivalence margin: -5 to 5 pg/mL. Judgments reproduce the final all-low-risk scenario.*

| **Comparison** | **N of studies** | **Within-study bias** | **Reporting bias** | **Indirectness** | **Imprecision** | **Heterogeneity** | **Incoherence** | **Confidence rating** |
| --- | --- | --- | --- | --- | --- | --- | --- | --- |
| CT vs CAE | 2 | No concerns | Low risk | No concerns | Some concerns | Some concerns | Some concerns | Low |
| CT vs HIT | 3 | No concerns | Low risk | No concerns | No concerns | Major concerns | Major concerns | Very low |
| CAE vs HIT | 0 | No concerns | Low risk | No concerns | Major concerns | No concerns | Major concerns | Very low |

Note: CT, conventional therapy; CE, combined aerobic and resistance exercise; CAE, continuous aerobic exercise; TC, Tai Chi; RE, resistance exercise; HIT, high-intensity interval training.

**Supplementary Table S11. CINeMA confidence assessment for Tumor necrosis factor-alpha (TNF-α)**

*Random-effects network meta-analysis; MD; equivalence margin: -5 to 5 pg/mL. Judgments reproduce the final all-low-risk scenario.*

| **Comparison** | **N of studies** | **Within-study bias** | **Reporting bias** | **Indirectness** | **Imprecision** | **Heterogeneity** | **Incoherence** | **Confidence rating** |
| --- | --- | --- | --- | --- | --- | --- | --- | --- |
| CT vs CAE | 1 | No concerns | Low risk | No concerns | No concerns | Major concerns | Major concerns | Very low |
| CT vs HIT | 3 | No concerns | Low risk | No concerns | No concerns | Major concerns | Major concerns | Very low |
| CAE vs HIT | 0 | No concerns | Low risk | No concerns | Major concerns | No concerns | Major concerns | Very low |

Note: CT, conventional therapy; CE, combined aerobic and resistance exercise; CAE, continuous aerobic exercise; TC, Tai Chi; RE, resistance exercise; HIT, high-intensity interval training.

**Supplementary Table S12. Conventional GRADE assessment for CRP and ESR**

*CRP and ESR were assessed using conventional GRADE because their evidence did not form connected treatment networks suitable for CINeMA.*

| **Comparison** | **N of studies** | **Within-study bias** | **Reporting bias** | **Indirectness** | **Imprecision** | **Heterogeneity** | **Incoherence** | **Confidence rating** |
| --- | --- | --- | --- | --- | --- | --- | --- | --- |
| CRP: Yoga and TC vs CT | 2 | Serious | Suspected | Serious | Serious | Serious | Not applicable | Very low |
| ESR: Yoga and TC vs CT | 2 | Serious | Suspected | Serious | Serious | Serious | Not applicable | Very low |

Note: For CRP and ESR, GRADE terminology is used. Risk of bias, inconsistency, indirectness, imprecision, and suspected publication bias were considered. Incoherence is not applicable to pairwise meta-analysis. CRP, C-reactive protein; ESR, erythrocyte sedimentation rate.
